# Genotyping and Expression Analysis of the Non-Truncated *LcELF3* Gene Shows a Link with Early Flowering in Lentil (*Lens culinaris* Medik.)

**DOI:** 10.3390/plants15203147

**Published:** 2026-10-15

**Authors:** Marzhan Kuzbakova, Gulmira Khassanova, Satyvaldy Jatayev, Baurzhan Arinov, Crystal Sweetman, Colin L. D. Jenkins, Kathleen L. Soole, Yuri Shavrukov

**Affiliations:** 1 Institute of Agronomy and Forestry, Saken Seifullin Kazakh Agrotechnical University, Astana 010000, Kazakhstan; m.kuzbakova@kazatu.edu.kz (M.K.); s.jatayev@kazatu.edu.kz (S.J.); b.arinov@kazatu.edu.kz (B.A.); 2 College of Science and Engineering, Biological Sciences, Flinders University, Adelaide, SA 5042, Australia; crystal.sweetman@flinders.edu.au (C.S.); colin.jenkins@flinders.edu.au (C.L.D.J.); kathleen.soole@flinders.edu.au (K.L.S.)

**Keywords:** allele-specific qPCR (ASQ) markers, *Early Flowering 3* (*ELF3*) gene, *Flowering Locus T* (*FT*) gene, flowering time, gene expression, haplotype, lentil, plant genotyping

## Abstract

Lentil is an important legume crop that exhibits substantial variation in flowering time (FT). This trait is controlled by a complex but well-characterized regulatory system in many plant species, in which the *Early Flowering 3* (*ELF3*) gene functions as a repressor of the *Flowering Locus T* (*FT*) gene. In the present study, two distinct genes, *LcELF3a* and *LcELF3b*, were identified in lentil. Sequencing of the *LcELF3a* gene confirmed the absence of the *elf3* mutation in studied accessions, which causes a truncated polypeptide and a very early-flowering phenotype in lentil plants. Natural genetic variation, consisting of six SNPs and one insertion/deletion (InDel), but lacking the *elf3* mutation, was grouped into two haplotypes, A and B, which were significantly associated with late and early flowering time, respectively. Genotyping using the newly developed ASQ marker LcELF3a-SNP3 revealed clear allele discrimination in both a lentil germplasm collection and hybrid breeding lines, identifying the homozygous genotypes *aa* and *bb*. The genotyping results with the LcELF3a-SNP3 marker confirmed that lentil accessions were associated with either late or early flowering time. Gene expression analysis using RT-qPCR in hybrid breeding lines and their parental genotypes showed high transcript levels of the *LcELF3a* and *LcFTa1* genes. Expression of the *LcELF3a* gene gradually declined, while flowering initiation progressed concomitantly with the upregulation of *LcFTa1* transcript accumulation. The expression of *LcELF3b* followed a similar pattern to that of *LcELF3a*, although at levels approximately four times lower. None of the other three *LcFT* genes (*LcFTa2*, *LcFTb1/b2*, and *LcFTc*) showed significant associations with flowering time in the hybrid breeding lines and parental genotypes examined. Seed yield was significantly higher in early FT cv.Krapinka and in late FT parent Flip92-36L, and in corresponding breeding lines, with *aa* and *bb* genotype of *LcELF3a*, respectively, whereas no differences were found between early- and late-flowering genotypes in the lentil germplasm collection. Other traits associated with adaptation to local environmental conditions are likely to have a greater influence on seed yield in lentil.

## 1. Introduction

The transition from the vegetative to the reproductive stage and the regulation of flowering time represent some of the most important processes in plant development. Flowering time is controlled by a complex genetic regulatory network that integrates internal plant developmental signals with environmental factors, including photoperiod, temperature, and moisture availability [1,2,3]. Flowering time is a component of this genetic system that determines the early or late onset of flowering. To do so, signals from key flowering integrators in major regulatory pathways must be combined to ensure the transition of plants to the reproductive stage [4]. This process has been optimised during the evolution of plant species to prevent excessively early or late flowering and is tightly regulated in response to environmental conditions [5,6], including legumes [7]. Therefore, flowering time is a critical component of plant development, involving numerous genes, including *Flowering Locus T* (*FT*), *Early Flowering 3* (*ELF3*), and other loci identified through genome-wide association studies (GWAS) and QTL analyses [8,9,10].

The *ELF3* gene is a transcriptional regulator that controls plant development through the circadian clock in response to light changes, and this gene is an important part of the time-to-flowering trait [11]. The *ELF3* gene has been extensively characterised in several legume crops. In pea, two duplicated gene family members, *ELF3a* and *ELF3b*, were identified and analysed, and their expression was shown to be coordinated with *FT* genes [12]. The function of the *ELF3* polypeptide was shown as a flowering repressor, preventing an excessively early start of flowering. It was reported that *ELF3* protein suppresses flowering under non-inductive photoperiods by binding and blocking production of gibberellin and *FT1* expression in the example of barley [13]. As *ELF3*-mediated repression decreases, *FT1* expression increases, thereby accelerating floral transition. This process was well-described in recent reviews [14], including in *Arabidopsis* as a model plant species [11].

In transcriptomic analysis of *Medicago truncatula*, one of the identified genes was *MtELF3* (LOC11431402), highly expressed in different developmental stages related to time to flowering [15]. In the annual legume plant species guar, *Cyamopsis tetragonoloba* L., expression of the *CtELF3* gene was strongly up-regulated in short-day (SD) conditions [16].

Lentil (*Lens culinaris* Medik.) exhibits substantial variation in growing season length and flowering time, traits that largely determine its adaptation to diverse agro-climatic conditions [17], and *LcELF3a* has been shown to function in coordination with *LcFT* genes involved in the regulation of early flowering [12,18]. A cluster of two genes, *LcFTa1* and *LcFTa2*, was identified in lentil chromosome LG6 during QTL analysis for days to flowering in mapping populations [19], and by using Diversity array technology, DArT-seq [20]. Both genes were highly expressed, but *LcFTa1* was primarily associated with an early-flowering phenotype in lentil genotypes [20]. Another cluster containing *LcFTb1* and *LcFTb2* was identified on linkage group LG6 and its association with early flowering was demonstrated using KASP markers [21].

More recent studies have confirmed that the genetic control of flowering time in lentil is strongly influenced by environmental conditions. For example, a major QTL ‘qDTF6.I’ was identified on chromosome 6, accounting for 23–56% of the phenotypic variation in flowering time, and it includes *LcFT* with further confirmation of the key role of the *FT* gene family in regulating the transition to flowering in lentils [22].

In lentil accession L4710 and cv. Precoz from Argentina, mutant *elf3* was reported as one SNP, replacing ‘G’ with ‘A’ nucleotide in the last position in exon 3 of the *LcELF3a* gene. This mutation produced a truncated polypeptide resulting in very early flowering and photoperiod-insensitive development [23]. Additionally, the *elf3* mutant has a deletion of one triplet, GAC, in the second exon. However, this mutation was relatively rare. For example, no Indian lentil genotypes contained this mutation, and they had different *LcELF3a* alleles showing various haplotypes resulting in a range of plants with early and late flowering time [24]. Similar results confirming the *elf3* mutation in cv. Precoz and in four accessions from Syria were reported later. The authors also identified a single Australian cv. Materno as containing the same *elf3* mutation, but it started flowering later than others [21].

In contrast, the very late-flowering Globe Mutant was studied in a cross with early-flowering lentil accession L4775 containing the *elf3* mutation [18]. The authors reported clear segregation for FT in 231 hybrid lines, and groups with early and late bulks were sequenced with a QTL-Seq approach, where significant genetic polymorphism was reported in the *LcELF3a* gene. The early-flowering hybrid lines were confirmed to contain the *elf3* mutation originating from the L4775 parent, whereas the late-flowering Globe Mutant had three deletions in the *LcELF3a* gene causing a very late-flowering habit [18].

Genetic variation in the *LcELF3a* gene consists of SNPs and insertion/deletion (InDel) polymorphisms organized into several haplotypes that are associated with flowering time [18,21,25]. In chickpea, SNP and haplotypes in the *CaELF3* gene were identified using whole genome shotgun (WGS) re-sequencing of two near-isogenic lines (NIL) with contrasting phenotyping results in plants with early and late start of flowering [26].

In soybean, the genetic system suppressing flowering is more complicated and includes three genes. The mutation *e6^PG^*, with an insertion in the first gene *J*, the ortholog of *ELF3*, resulted in the new *E6* allele. This mutated *E6* allele did not suppress the second flowering repressor *E1* and, therefore, expression of the *FT* gene remains inhibited. The mutant *e6^PG^*, therefore, had a delay in flowering accompanied by higher yield under SD conditions [27]. In *Arabidopsis thaliana*, three haplotypes or haplogroups were identified based on SNP in *AtELF3*, which were associated with seasonal temperature during plant growing. One of the SNPs was located within intron 2 of *AtELF3* and this SNP2 affected the expression of the truncated ELF3β transcript. Additionally, it was shown that this SNP2 in *AtELF3* had a functional effect in *Arabidopsis* genotypes responding to the changes in growing conditions [28].

Many publications about lentil have reported that early-flowering and -maturing plants produced higher seed yield (a negative correlation between FT and seed yield) [29,30,31,32]; a similar dynamic is reported in pigeon pea, *Cajanus cajan* L. [33]. However, this relationship was not universal among legumes and, in contrast, other publications reported preferable late-flowering genotypes (a positive correlation between flowering time and seed yield). For example, in chickpea, strong positive correlations were reported between phenotyping and genotyping associations of FT and seed yield [34,35]. Nevertheless, some studies have found no significant relationship between flowering time and seed yield [36], whereas others have demonstrated that this relationship is highly dependent on environmental conditions and management practices [37,38].

To date, studies have mainly focused on lentil accessions from Australia [21], ICARDA germplasm collections, and recombinant inbred line (RIL) populations originating primarily from ICARDA and India [9,20,24]. However, comprehensive studies examining flowering-time diversity and the molecular structure of the *LcELF3* gene, together with its relationship to other flowering-related genes, are lacking in lentil germplasm adapted to the environmental conditions of Kazakhstan. We hypothesized that local lentil accessions and hybrid breeding lines from Kazakhstan contain diverse genotypes with previously undescribed genetic variation in the *LcELF3* gene.

To address this knowledge gap, the following objectives were investigated: (1) conduct a molecular characterisation of the *LcELF3a* gene using a genotyping approach; (2) identify *LcELF3a* haplotypes and evaluate their association with flowering time; (3) perform a comprehensive expression analysis of *LcELF3* and *LcFT* genes; and (4) assess the relationship between flowering time and seed yield in local lentil accessions and hybrid breeding lines.

## 2. Materials and Methods

### 2.1. Plant Material and Trait Evaluations

Seeds of lentil (*Lens culinaris* Medik.) accessions were provided by S. Seifullin Kazakh AgroTechnical Research University (KATRU), Astana (Kazakhstan); Vavilov Research Institute of Plant Genetic Resources (VRIPGR), Saint Petersburg (Russia), and from a local breeding collection. The full list of the 66 lentil germplasms studied is presented in Supplementary Material S1. The choice of the lentil accessions was based on initial observation and scores during preliminary experiments in the hybridisation program.

Two hybrid populations were produced by the authors: (1) ♀ Flip92-36L × ♂ ILL-1552, and (2) ♀ Krapinka × ♂ Flip96-48L. Three of the parents originated from the ICARDA germplasm collection: two of them, Flip92-36L and Flip96-48L, from Syria, whereas accession ILL-1552 originated from Iran. The fourth parent was a local cv. Krapinka from Kazakhstan [39]. Originally, 10 F_2_ breeding lines were developed in each population, and at a later stage, three and 10 F_4_ breeding lines from each population were selected for qPCR analysis and for the field trial, respectively, as the most suitable for growing in the local environment.

Eight lentil accessions were selected for Sanger sequencing, four of which were parents of two hybrids, indicated above. The four other accessions included two cultivars, Vekhovskaya and Niva-95, from Russia, and two accessions, IG-70915 Syrian local landrace and IG-69812 Lebanese local landrace, from Syria and Lebanon, respectively.

For molecular experiments with parents and hybrids, seeds were sown in 20 cm diameter pots with 3.0 kg of soil mix with equal volumes of commercial potting mix and soil from a research field near KATRU (Kazakhstan). Plants were grown at KATRU Campus during the summer season on the same time schedule as in the research fields. Pots were watered twice weekly on a portable scale, keeping the soil moisture level consistent at 80% field capacity.

**Figure 1:**
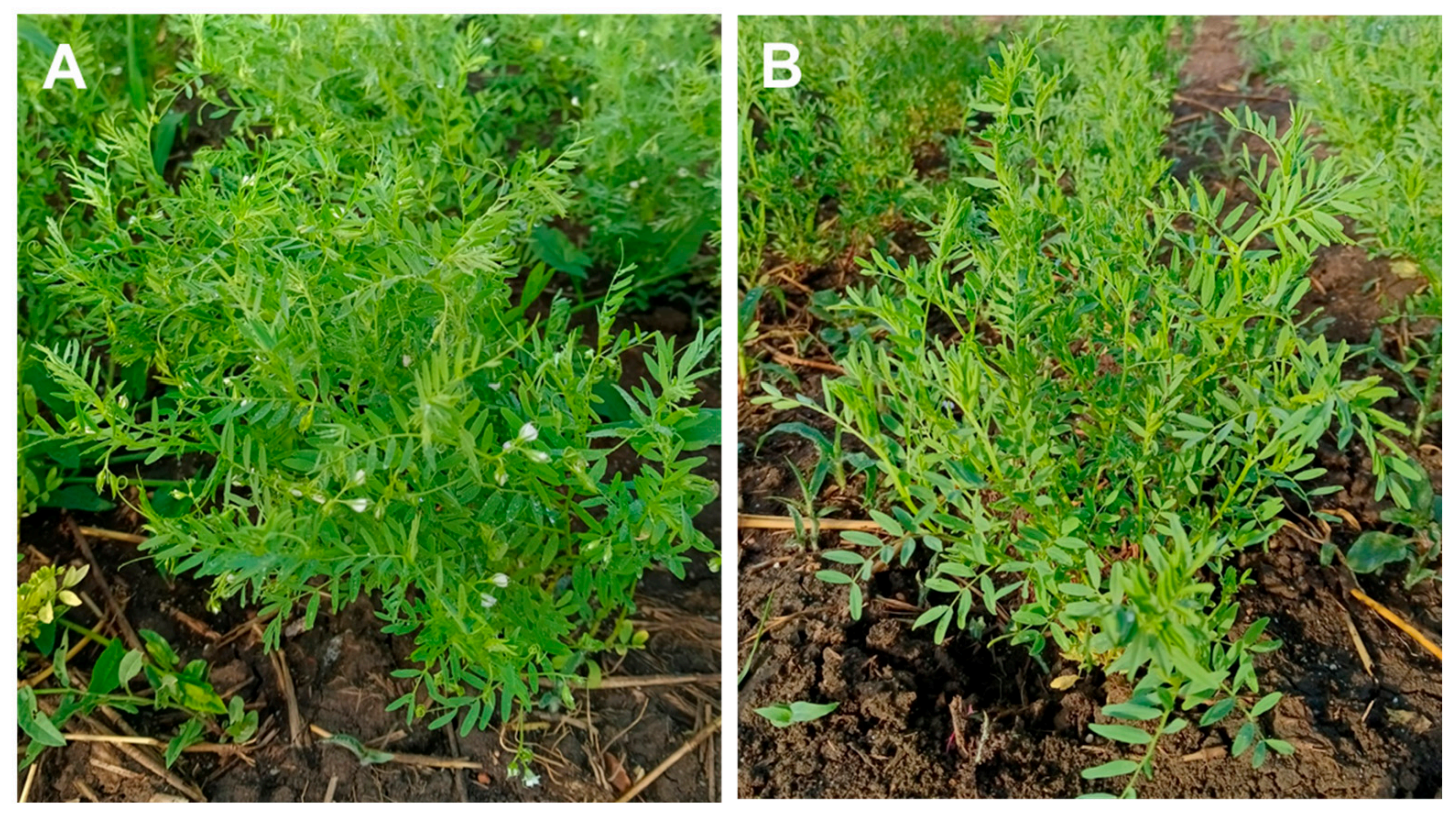
Example of lentil accessions with early flowering (**A**): cv. Krapinka (Kazakhstan); and late flowering habit (**B**): Flip92-36L (Syria). Seeds were sown on the same day with similar dates for ger-mination and seedling occurrence. Plants were grown in the research field near KATRU, Astana (Kazakhstan) in 2025.

For flowering time trait (FT) and seed yield evaluation in the lentil germplasm collection and in parents and hybrids, plants were grown in the research field near KATRU, in the Akmola region, Northern Kazakhstan, over three years (2023–2025), from where the soil was taken for pots, as described above. An example of plants with early and late FT is presented in Figure 1.

Each genotype was grown in a single row, 4 m in length, 10 cm between plants in a row, and 60 cm between rows with three fully randomised replicates. Weather conditions were exactly the same as in our published earlier paper [40].

In the current study, FT trait was scored per plot and counted as the number of days recorded from about 50% seedling occurrence until about half the plants started flowering in each plot. Seed yield was weighed after harvesting of all plants from each plot and re-calculated for g/m^2^. Data from plots with low plant density were discarded from further analysis.

### 2.2. Identification of LcELF3 Genes in Lentil

The sequences of two *LcELF3* genes in lentil were retrieved from the database of PCD, Pulse Crops Database [41], using NCBI sequence accession JX946295 for *LcELF3a* in the published report [12]. This *LcELF3a* sequence was used in initial and final searches of the database GenomeNet-BLASTN and BLASTP, respectively, in Kyoto University Bioinformatics Center, for homologs in closely related legumes [42]. The threshold cut-off Expectation value (E-value) was set as 1 × e^−^^100^ for selection of candidate genes for homologs of *ELF3a* and *ELF3b* from other legumes using BLAST analysis in the used databases. This allowed identification of exactly required homologs while avoiding genes with partial similarity of sequences.

Chromosome locations, positions on the physical map and *LcELF3* gene identifications in lentil were based on PCD, whereas those for other legumes were found either in PCD or in LIS (Legume Information System) [43]. All sequences of encoded proteins from the identified *ELF3* genes in the studied legume species were downloaded from GenomeNet, PCD and LIS databases for further molecular phylogenetic analysis and are presented in Supplementary Material S2.

The molecular–phylogenetic dendrogram was constructed based on amino acid sequences of polypeptides using CLUSTALW, ver.1, Multiple Sequence Alignment at GenomeNet Database Resources [44]. The results file was converted into a ‘nex’ file for further use in SplitsTree4, version 4.14.4, from algorithms in the bioinformatics website at the University of Tübingen, Germany [45]. The algorithm used for SplitsTree4 analysis was ‘NJ’ with network ‘NeighbourNet’, and Bootstrap value was not included in the phylogenetic tree [45]. For further sequencing and genotyping analyses, only one *LcELF3a* was selected as the confirmed most important gene controlling FT in various plant species, as described in the Section 1.

### 2.3. DNA Extraction, PCR, Sequencing, and SNP and InDel Identification

Two to three young leaf samples were collected in 10 mL tubes from individual plants and frozen in liquid nitrogen. DNA was extracted using the CTAB method as described earlier [46] with minor modifications. Frozen leaf samples were ground with ball bearings and a Vortex mixer, keeping samples frozen, as described for RNA extraction. The washed and dried DNA pellet was finally dissolved in 100 µL of 1/10 diluted TE Buffer with 25 µg of RNase A added. The DNA concentration was measured by NanoDrop spectrophotometer (Thermo Fisher Scientific, Waltham, MA, USA), and DNA quality was assessed on a 1% agarose gel.

The primers were designed based on the DNA sequence of *LcELF3a* from the *L. culinaris* reference genome of cv. Redberry, targeting two genetic fragments: (F1-R1) in the second exon and intron, 863 bp, and (F2-R2) from second intron to fourth exon, 853 bp. The sequences of the primers as well as the entire *LcELF3a* (Lcu.2RBY.3g063730) gene are presented in Supplementary Materials S3.

Regular PCR conditions were used, as described earlier [47], with some modifications for sequencing. Briefly, the total reaction volume was increased to 60 μL and contained 6 μL of template lentil DNA (20 ng/mL) with the following components in their final concentrations for each reaction: 1×SE PCR buffer including 1.5 mM MgCl_2_, 0.2 mM of each dNTPs, 0.25 mM of each primer, and 1.0 U of E332 Taq DNA polymerase (SibEnzyme, Novosibirsk, Russia). PCR was conducted on a SimpliAmp Cycler (Thermo Fisher Scientific, Waltham, MA, USA) using a program with the following steps: initial denaturation, 94 °C for 2 min; 35 cycles of 94 °C for 15 s, 55 °C for 15 s, and 72 °C for 1 min; and a final extension of 72 °C for 3 min.

The PCR products were purified using a PCR Purification kit (Syntol, Moscow, Russia) following the manufacturer’s instructions, and their concentrations were measured using a NanoDrop spectrophotometer (Thermo Fisher Scientific, Waltham, MA, USA). Sanger sequencing was carried out using BigDye Terminator v.3.1 reagents and SeqStudio, Applied Biosystems (Thermo Fisher Scientific, Waltham, MA, USA) at the Research Platform for Agricultural Biotechnology, S. Seifullin KATRU, Astana (Kazakhstan). SNPs and InDels were identified using manual comparison of the visualized sequences using the Chromas computer software program, ver.2.0. The identified SNPs were verified by sequencing of the same PCR products in both directions.

### 2.4. ASQ Plant Genotyping

The ASQ method was used for plant genotyping based on the previously published protocol [48] with the following modifications. The molecular probes with a short 4-bp tag were used, and two allele-specific forward primers together with one reverse primer were designed for SNP, LcELF3a-SNP3, to designate haplotypes LcELF3a–HapA and –HapB. The composition of the PCR cocktail for ASQ genotyping was the same as published earlier [49], and sequences of the allele-specific primers are presented in Supplementary Material S4.

The primers and molecular probes were obtained from DNA Synthesis (Moscow, Russia). Each reaction had a 10 μL cocktail in total and was loaded in a 96-well microplate. Allele discrimination was determined using two types of instruments, QuantStudio-7 Real-Time PCR system instrument (Thermo Fisher Scientific, Waltham, MA, USA) at KATRU and CFX96 Real-Time qPCR system (BioRad, Hercules, CA, USA) at Flinders University with automatically recorded fluorescence. Amplification of FAM and HEX was checked and controlled, whereas genotyping results were determined in a post-run step with analysis of Real-Time dRn setting using the inbuilt software for the Thermo Fisher Scientific instrument and with Maestro software (ver.2.3) for the BioRad instrument. Genotyping experiments were carried out with three individual plants (biological replicates) and two technical repeated runs for each lentil genotype.

### 2.5. RNA Extraction, cDNA Synthesis and RT-qPCR Gene Expression Analysis

Leaf samples were collected from lentil plants at three stages: (1) three-week-old plants designated as ‘vegetative’ or ‘before flowering’; (2) plants carrying their first three flowers, designated as ‘flowering’; and (3) three weeks after the initiation of flowering, regarded as ‘seed development’. Four biological replicates from each genotype of parents and hybrid lines described in Section 2.1 were randomly selected and leaves snap-frozen in liquid nitrogen and stored at −80 °C. Leaf samples were ground with two 8 mm stainless ball bearings using a Vortex mixer, keeping the samples frozen.

RNA was extracted following the previously published protocol [50] with modification using PureZOL reagent (BioRad, Hercules, CA, USA) following the manufacturer’s protocol. Finally, the RNA pellet was dissolved in 100 µL of elution buffer, measured for quantity using a NanoDrop spectrophotometer (Thermo Fisher Scientific, Waltham, MA, USA), and RNA quality and integrity were checked in 1% agarose gel. Each sample with 2 μg of RNA was reverse transcribed using OT-M-MuLV-RH Reverse Transcriptase kit with supplementary DNase treatment (BiolabMix, Novosibirsk, Russia).

Produced cDNA samples were diluted with sterile water (1:10) and used for qPCR analyses with a QuantStudio-7 Real-Time PCR system instrument (Thermo Fisher Scientific, Waltham, MA, USA), following the qPCR protocol described earlier [51] with some modifications. The total volume (10 μL) of qPCR in each well included 5 μL of 2×Biomaster HS-qPCR SYBR Blue (BiolabMix, Novosibirsk, Russia), 4 μL of diluted cDNA, and 1 μL of two gene-specific primers (3 μM of each primer) as per the manufacturer’s recommendation.

Thermal cycling conditions included a brief initial melt at 95 °C for 3 min, followed by 40 cycles of 95 °C for 5 s and 60 °C for 20 s, and finished with a melt curve from 60 °C to 95 °C increasing by 0.5 °C increments every 5 s. The efficiencies of all qPCR primers were calculated based on the slope of the corresponding calibration line, and their suitability was confirmed. Specificities of target and reference gene amplifications were verified with single distinct peaks on a melting curve and a single band of the expected size in a 2% agarose gel.

Seven target genes were studied: (1) *LcELF3a* (Lcu.2RBY.3g063730); (2) *LcELF3b* (Lcu.2RBY.5g070480); (3) *LcFTa1* (Lcu.2RBY.6g043850); (4) *LcFTa2* (Lcu.2RBY.6g043870); (5) *LcFTa3* (Lcu.2RBY.2g046590); (6) *LcFTb1-b2*, in bulk for two genes (*-b1* and *-b2*) together due to very small differences in their sequences (Lcu.2RBY.6g000730 and 6g000760); and (7) *LcFTc* (Lcu.2RBY.6g043940). Expression data for all target genes were calculated with normalisation of gene expression relative to the internal reference gene *LcElf4A-III*, Eukaryotic initiation factor 4A-III (Lcu.2RBY.2g019770), and stability of the reference gene was validated. Stability and specificities of reference and target gene amplifications were verified with single distinct peaks on a melting curve and a single band of the expected size in a 2% agarose gel. Primer sequences with amplicon sizes used for qPCR are presented in Supplementary Material S5. At least four biological replicates and two technical repeats were used for each genotype and time-point in qPCR experiments.

The Relative standard dilution method was used based on the ABI Guide for relative quantitation of gene expression using RT-qPCR [52], where serial dilutions were applied for each target and reference gene individually. Threshold cycle values were determined based on linear calibration of template cDNA dilution factor and Cq value.

### 2.6. Statistical Treatment

Means, standard errors and significance levels were calculated using the software package of Excel 365, Microsoft. Student’s *t*-test for two samples with equal variances was used for comparison of FT trait and seed yield in hybrid breeding lines and germplasm collection. Single factor ANOVA with post-hoc Tukey test was used for gene expression analyses including biological replicates (independent plants) as non-interacted factors with residual diagnostics and confidence interval for significant differences. Kruskal–Wallis test was used to estimate statistical differences between genotype groups with corresponding phenotypes. Pearson’s correlation was used for testing FT and seed yield associations. For field trials, three fully randomised plots were used as replicates over three years. For molecular analyses, three and four biological replicates (individual plants) were used for ASQ genotyping and gene expression analyses, respectively, with two technical repeats (instrumental runs) in both types of experiments, for each genotype and experiment.

## 3. Results

### 3.1. Molecular Phylogenetic Analysis of LcELF3 Genes in Lentil and Other Legumes

**Figure 2:**
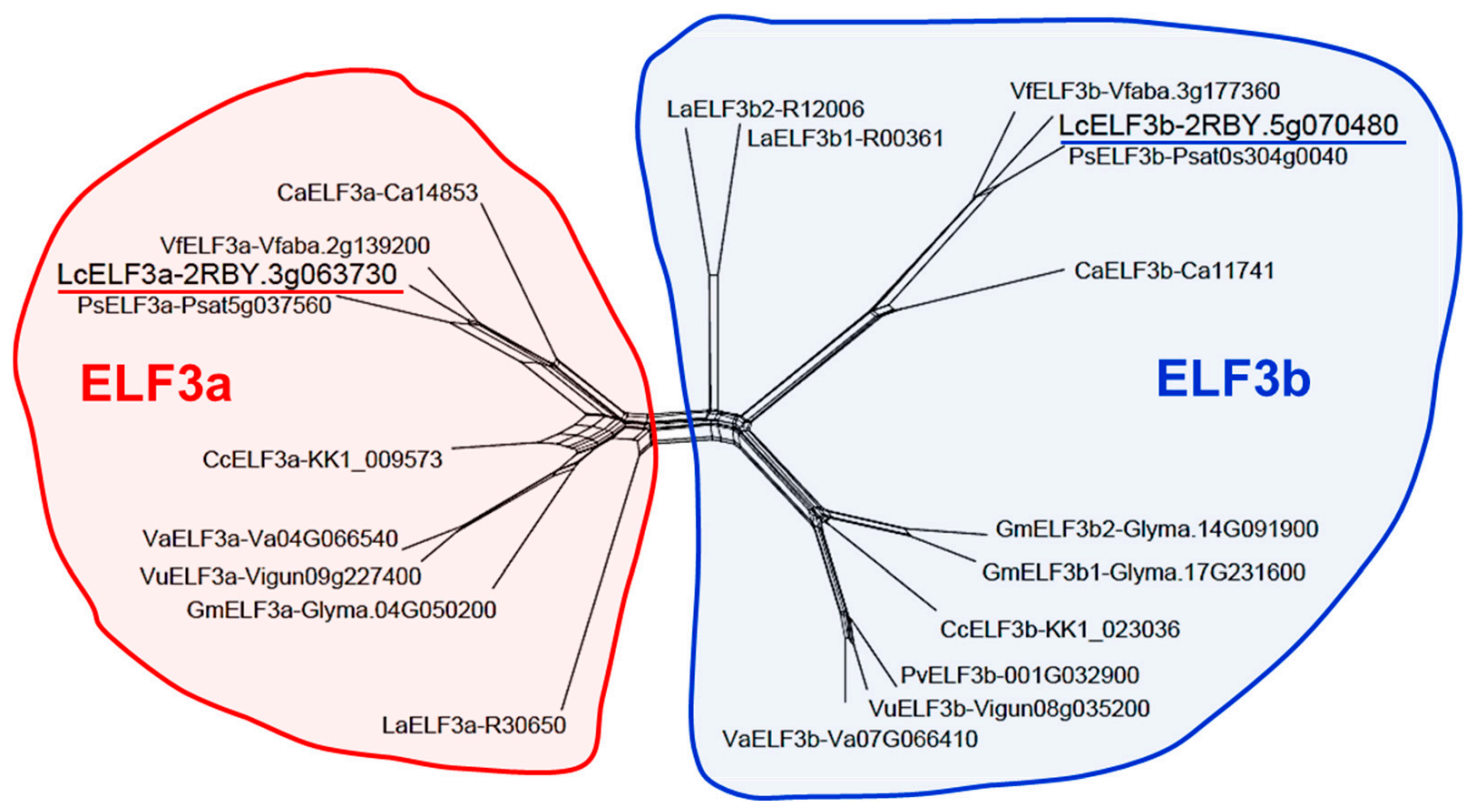
Molecular phylogenetic tree for *ELF3* genes in lentil and closely related legumes based on amino acid sequences of encoded polypeptides. Two clades for *ELF3a* and *ELF3b* are designated by red and blue colour, respectively. Names of the genes and their identification in the genome correspond to the legume species: chickpea, Ca, *Cicer arietinum* L.; pigeon pea, Cc, *Cajanus cajan* (L.) Huth.; soybean, Gm, *Glycine max* (L.) Merr.; narrowleaf or blue lupin, La, *Lupinus angustifolius* L.; lentil, Lc, *Lens culinaris* Medik.; pea, Ps, *Pisum sativum* L.; common bean, Pv, *Phaseolus vulgaris* L.; adzuki bean, Va, *Vigna angularis* (Willd.) Ohwi & H.Ohashi; faba bean, Vf, *Vicia faba* L.; and cowpea, Vu, *Vigna unguiculata* (L.) Walp. *LcELF3* genes are indicated by larger text and underlined in colour. The molecular dendrogram was constructed using the SplitsTree4 program [45]. The sequences of encoded ELF3 proteins in the studied legumes are presented in Supplementary Material S2.

Based on publicly available lentil genome data in *Lens culinaris*, cv. Redberry, version gnm2.ann1 [41], two genes, *LcELF3a* (Lcu.2RBY.3g063730) and *LcELF3b* (Lcu.2RBY.5g070480), were identified. Their encoded polypeptides showed similarity in phylogenetic analysis to pea and soybean, chickpea, lupin, and other closely related legumes (Figure 2).

The first clade of the dendrogram, with the *ELF3a* group of genes, represents a single, non-duplicated copy of the gene in each plant species examined. In contrast, the second clade of the phylogenetic tree comprises duplicated *ELF3b* genes in soybean (*GmELF3b1* and *GmELF3b2*) and in lupin (*LaELF3b1* and *LaELF3b2*). Other species examined had a single copy of the *ELF3b* gene. The two genes studied in lentil, *LcELF3a* and *LcELF3b*, showed close similarity with faba bean and pea, moderately close with chickpea, but more greatly distanced from other legumes based on the encoded proteins (Figure 2). For further experiments, only one gene, *LcELF3a*, was selected.

### 3.2. Sanger Sequencing, SNP Identification in LcELF3a Gene in Lentil Accessions

During sequencing of the *LcELF3a* gene in the eight lentil accessions described, one genotype, IG-69812, a Lebanese local landrace, was found partially admixed and, therefore, it was dismissed from further study. Sequencing of the other seven genotypes was of good quality. The first important finding was that all studied lentil accessions were monomorphic in position 2614 from the ‘Start’ codon in genomic DNA (=position 2648 in the annotated sequence accession JX946295), the last position of exon 3. The nucleotide ‘G’ was present in that position in all genotypes with no transition to ‘A’. Additionally, in the sequencing of exon 2, all studied lentil accessions showed the presence of a ‘GAC’ triplet with no deletions. These results indicate the absence of the *elf3* mutation and the corresponding allele. Therefore, all lentil accessions examined, including parents of hybrid populations, had the original, non-truncated LcEFL3a polypeptide, and they occur as naturally variable flowering genotypes with early and late flowering.

**Figure 3:**
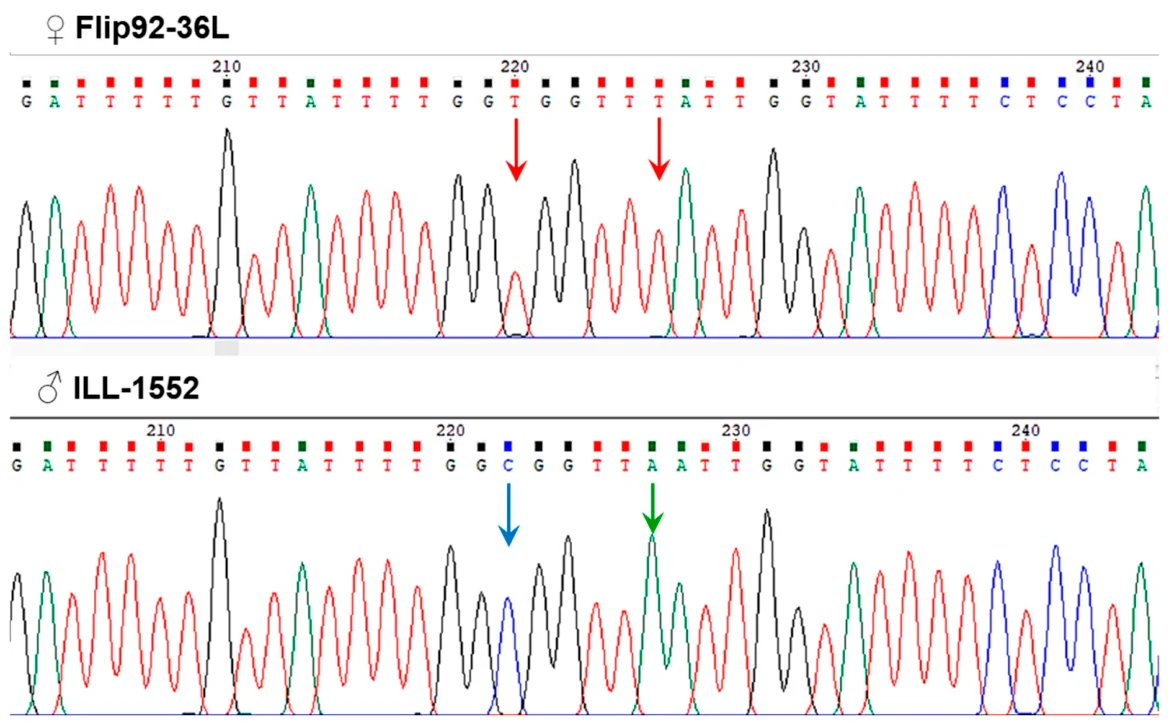
Fragments of Sanger sequencing of the second intron of the gene *LcELF3a* (Lcu.2RBY.3g063730) in two lentil accessions used as parents for hybridisation, maternal (Flip92-36L) and paternal (ILL-1552). Positions of two identified SNPs, SNP1 and SNP2, are shown by arrows with the corresponding colours.

With further sequencing of the *LcELF3a* gene, six SNPs and one InDel were identified in the second intron, in fragment F1-R1 of the gene (Figure 3 and Supplementary Material S6). However, the second fragment (F2-R2) was strongly conserved and no SNP was found in the fragment after sequencing of seven lentil accessions of diverse origins. Two haplotypes, A and B, were identified in this study comprising polymorphism in six SNPs and one InDel, linked in two separate groups. Four lentil genotypes, with haplotype A, represent the late-flowering phenotype; three of them originated from Syria, while the fourth accession, cv. Niva-95, had Russian origin. Additionally, the reference genotype cv. Redberry also shows the same *LcELF3a* haplotype A, based on the publicly available and fully sequenced genome. Three other lentil accessions with an early start of flowering had haplotype B, based on all six identified SNPs. These accessions include ILL-1552, and two cultivars, Krapinka and Vekhovskaya (Table 1 and Supplementary Material S6).

In addition, parents of two hybrids, ♀ Flip92-36L and ♂ ILL-1552, and ♀ Krapinka and ♂ Flip96-48L, were identified as polymorphic, representing haplotype A or B, respectively. Therefore, 10 and 6 selected F_4_ breeding lines, with their parents, from both hybrids, were used for *LcELF3a* genotyping of plants and further expression analyses of this and other associated *LcFT* genes.

### 3.3. Comparison of Data for Flowering Time in Two Haplotypes of the LcELF3a Gene

**Figure 4:**
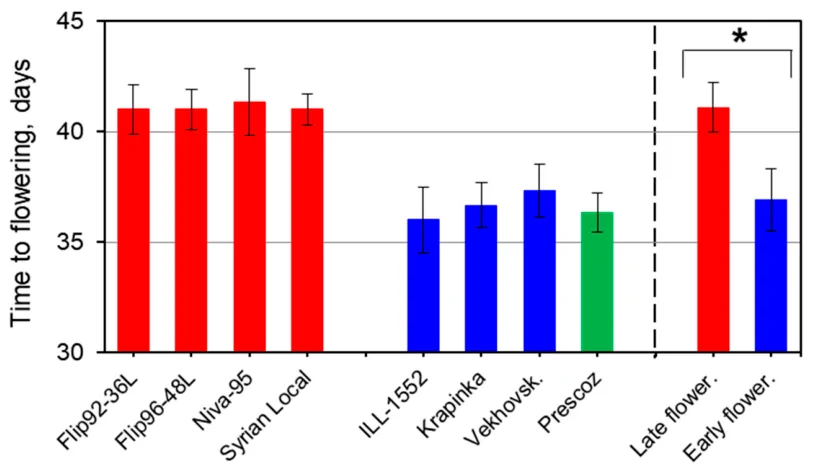
Evaluation of FT in lentil accessions with haplotype A (red bars) and haplotype B (blue bars) of the *LcELF3a* gene, shown as average data ± SE from three replicates, over three years in Northern Kazakhstan. Data for cv. Precoz (green bar) with mutation *elf3* were derived under the same conditions, shown separately. Significant difference (* *p* < 0.05) between the two groups is shown using *t*-test for two samples with equal variance.

Two groups of lentil accessions with identified haplotypes A and B of the *LcELF3a* gene were assessed for flowering time (Table 1). The data collected over three years of observation indicated a significant difference in the FT trait between two haplotypes comprising 4.4 days on average using *t*-test (*p* = 0.0453 < *p* = 0.05; *n* = 72) (Figure 4). Plants with haplotype A started flowering 41.1 days after germination, compared to 36.7 days for haplotype B. The lentil cv. Precoz also showed an early flowering time phenotype of 36.3 days after germination, evaluated over three years, indicating no difference to haplotype B for the trait. However, cv. Precoz contains mutation *elf3* and represents a distinct group of accessions compared to those examined here (Figure 4).

Based on the results for flowering time presented in Figure 4, the histogram of all 66 studied lentil accessions was prepared showing their distribution for days to flowering based on the average over three studied years (Supplementary Material S7). Groups of late- and early-flowering time had an average of 41.5 and 38.1 days from seedling occurrence to flowering, respectively. The distributions of late- and early-flowering accessions on the histogram overlapped at the point of 39.7 days and accounted for only two accessions, which were classified as ‘intermediate’ (Supplementary Material S7).

### 3.4. SNP Genotyping in Lentil Plants Using ASQ Molecular Markers for the LcELF3a Gene

**Figure 5:**
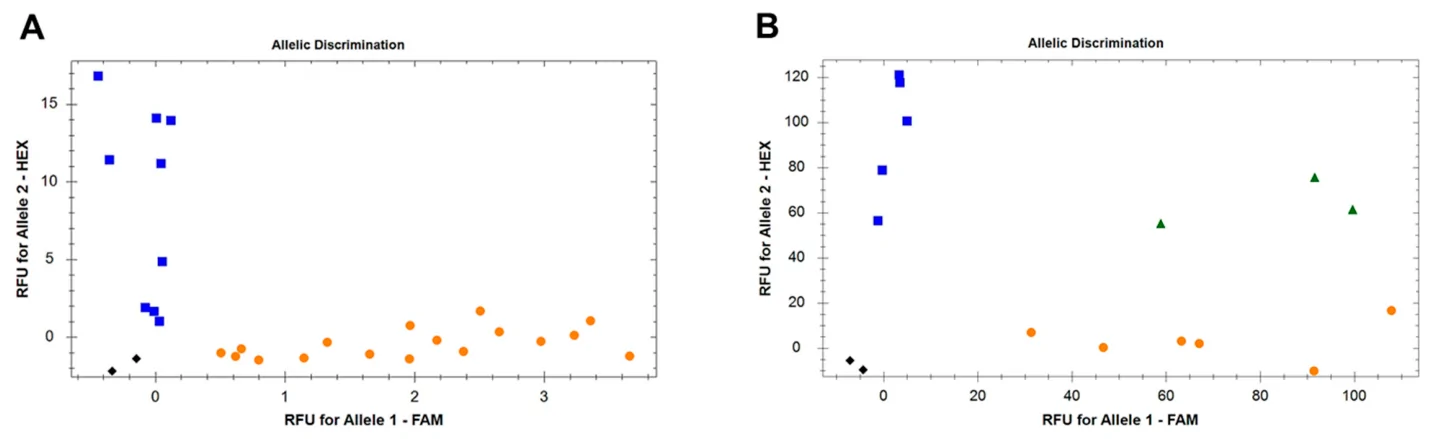
Example of plant genotyping for *LcELF3a* alleles in SNP3 in the lentil germplasm collection (**A**) and in two hybrids (**B**). The allele discrimination plot shows results of RFU (Relative fluorescence units) for FAM and HEX signals, assigned to X- and Y-axes of the figure using ASQ molecular marker LcELF3a-SNP3. Orange dots and blue squares represent homozygote genotypes *aa* and *bb* for alleles indicated by FAM and HEX, respectively. Green triangles indicate fluorescence signals of both fluorophores, FAM and HEX, determined as heterozygotes *ab*. Allele discrimination was based on dRn automatic SNP calling. The black rhombuses show no template control (NTC) using sterile water instead of template DNA.

ASQ molecular marker LcELF3a-SNP3 was developed targeting SNP3 in the *LcELF3a* gene and used to compare genotypes (Table 1 and Figure 5), confirming their *aa* and *bb* genotypes, respectively. Plants from a 66-member lentil germplasm collection, representing an independent validation panel, were genotyped using ASQ marker LcELF3a-SNP3, and clear discrimination of *LcELF3a* alleles of SNP3 was found (Figure 5A) with 31 and 26 homozygotes, *aa* and *bb*, respectively, and 9 heterozygotes *ab* (Supplementary Material S8).

Figure 5B shows *LcELF3a* allele discrimination in two hybrid populations, where parents Flip92-36L and Flip96-48L with genotypes *aa* and other parents, ILL-1552 and Krapinka, with genotype *bb*, were confirmed as regards their *LcELF3a* genotype status. The ten hybrid F_4_ breeding lines were clearly distributed with either parent of homozygous genotypes *aa* or *bb*, but some breeding lines remained heterozygous, *ab*, inheriting one allele from either parent. In summary, the ASQ marker LcELF3a-SNP3 successfully discriminated alleles in both the germplasm collection and in the studied hybrids.

### 3.5. Comparison of Genotyping and Phenotyping of Plants for FT

The lentil germplasm collection was examined for flowering time over three years and average FT is presented in Supplementary Material S8. Three groups were found, with most being classified as late or early FT (35 and 27 accessions, respectively), and four accessions with intermediate FT (Table 2). Using the Kruskal–Wallis test, highly significant differences were found during comparison between the genotypes of the three groups, *aa*, *ab* and *bb* (Table 1, Figure 4), and the corresponding phenotyping results for FT (Table 2).

In Table 2, ‘mismatches’ represent ‘predictive accuracy’ as differences between genotype and the expected corresponding phenotype; for example, between nine heterozygote *ab* genotypes and two intermediate FT, the difference equals seven ‘mismatches’. There were six mismatches for homozygotes *aa* and one mismatch for homozygote *bb*. The mismatches represent a level of overlapping, but the number is insufficient to diminish the significant association with the FT trait (Table 2). The primary genotyping and phenotyping data for the germplasm collection is presented in Supplementary Material S8, and statistical treatment details of the Kruskal–Wallis test are in Supplementary Material S9.

### 3.6. Differential Expression of LcELF3 and LcFT Genes During Development of Lentil Plants

Relative expression of the two genes, *LcELF3a* and *LcELF3b*, and four genes *LcFT*, which are involved in the control of the genetic network for the time-to-flowering trait in lentil, were determined using RT-qPCR. Six F_4_ breeding lines (designated 21/8 and 21/3, three for each of two hybrids) were selected for study, together with their parents from hybrid 1 [♀ Flip92-36L × ♂ ILL-1552] and hybrid 2 [♀ Krapinka × ♂ Flip96-48L], respectively.

**Figure 6:**
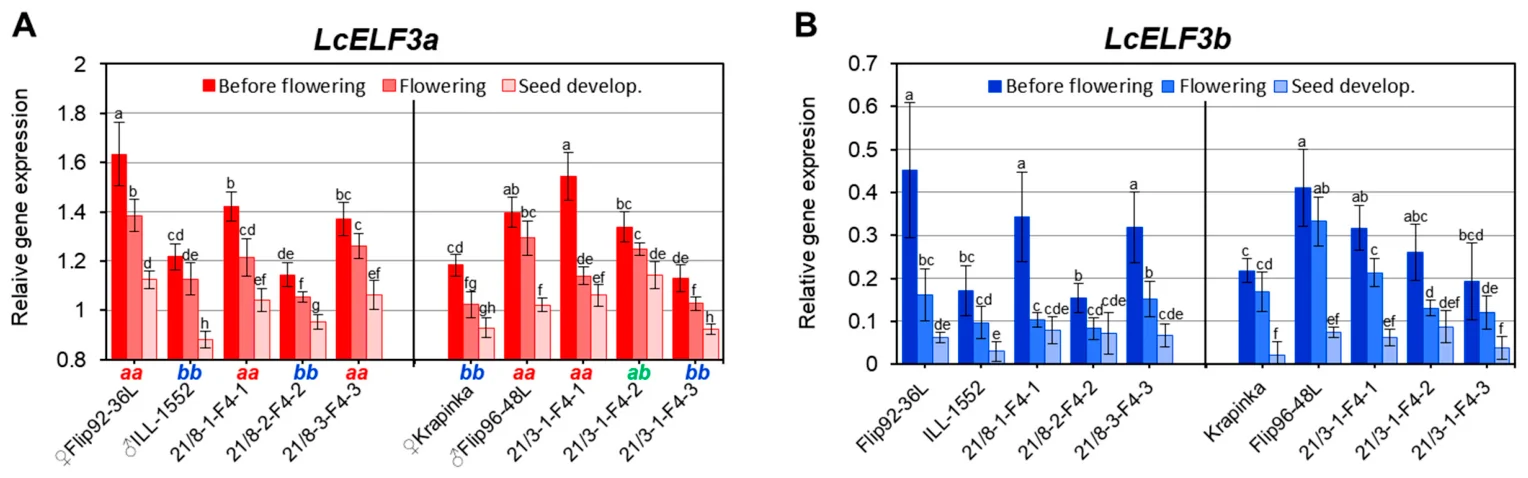
RT-qPCR expression analysis of *LcELF3a* gene (Lcu.2RBY.3g063730) (**A**) and *LcELF3b* (Lcu.2RBY.5g070480) (**B**) during plant development. Parents and F_4_ breeding lines from two hybrid populations during three developmental stages, indicated in the Figure. On the left-hand side of each panel, hybrid 1 parents, ♀ Flip92-36L and ♂ ILL-1552, are shown, with three breeding lines 21/8, while on the right-hand side, hybrid 2 parents, ♀ Krapinka and ♂ Flip96-48L, are shown, with three breeding lines 21/3. In *X*-axis, on panel (**A**), genotypes for ASQ marker LcELF3a-SNP3 are indicated by red, blue and green letters. Expression data were normalised using the internal reference gene, *LcElf4A-III* (Eukaryotic initiation factor 4A-III), and data are presented as the average ± SE of four biological replicates (individual plants), used as non-interactive factors, and two technical repeated runs for each genotype and developmental stage. Expression results for each parent are designated above the bars by different letters. Shared letters indicate the absence of difference within each hybrid population. Significant differences with confidence interval (*p* = 0.013 < 0.05; F = 3.273) are shown by different letters in a certain hybrid. Analysis used single-factor ANOVA with post-hoc Tukey test with residual diagnostics of initial data.

Expression analyses for *LcELF3* genes are present in Figure 6. All genotypes showed highest expression of the gene *LcELF3a* at the vegetative stage before flowering, ranging between 1.63 and 1.13 expression units relative to the reference gene. Highest apparent level of expression was found in parent Flip92-36L and breeding lines 21/8-1-F4-1 and -3 for hybrid 1, and in parent Flip96-48L and breeding lines 21/3-1-F4-1 and -2 for hybrid 2. Throughout plant growth and development, expression of *LcELF3a* gradually decreased in all genotypes, but gene expression differences were less consistent at flowering and seed development stages in some breeding lines (Figure 6A).

A similar trend was found in the expression of *LcELF3b*, but the level of expression was about four times smaller compared to *LcELF3a*, ranging between 0.45 and 0.15 expression units relative to the reference gene (Figure 6B). Therefore, genotypes *aa* with late transition to flowering were associated with higher expression of both *LcELF3* genes, but the greater level expression profile was recorded for *LcELF3a*. In contrast, genotypes *bb* with early flowering time showed lower mRNA levels of both *LcELF3* genes at all stages of development (Figure 6A,B).

**Figure 7:**
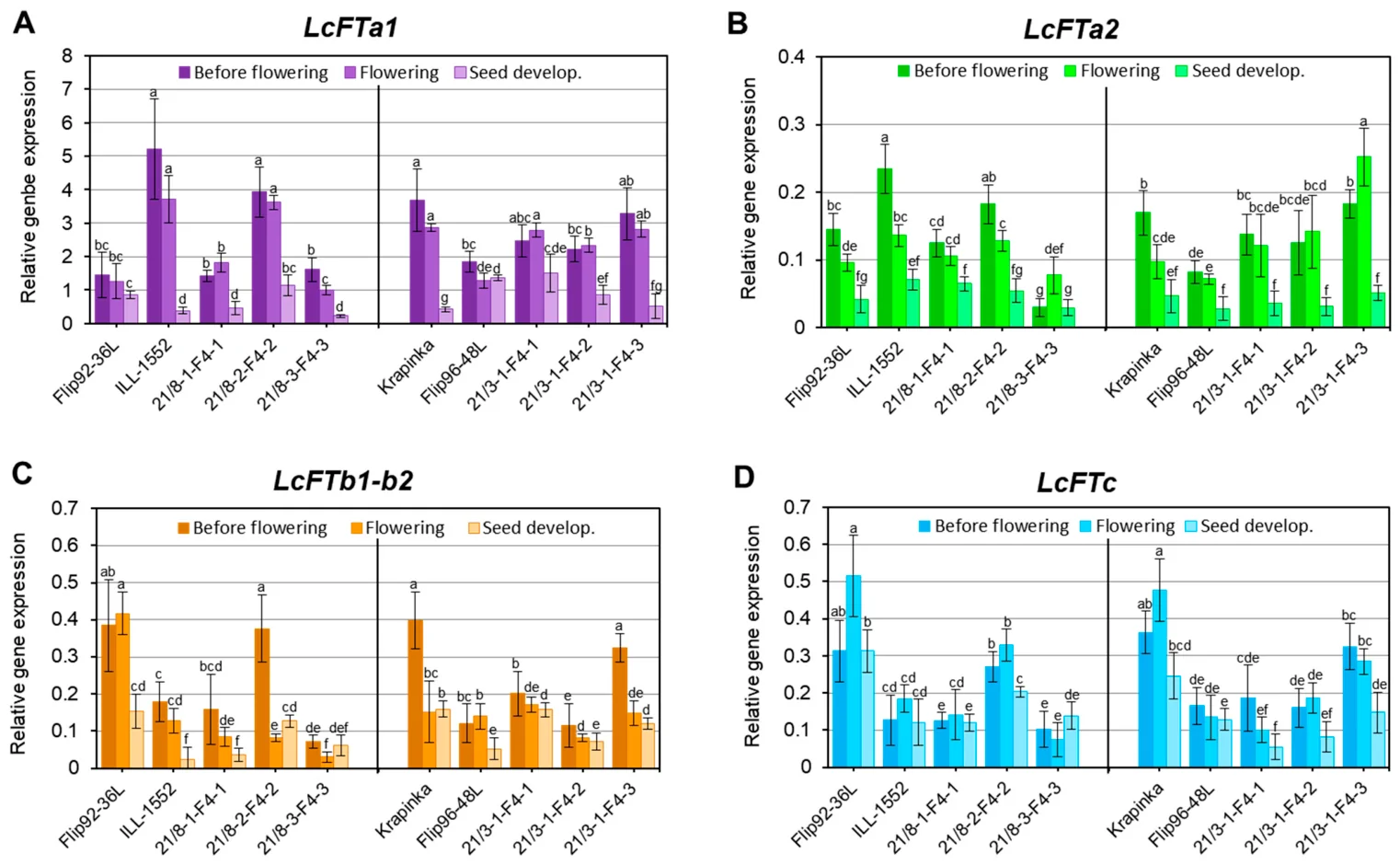
Expression analysis of four *LcFT* genes during vegetative–reproductive plant development, using RT-qPCR. Parents and F_4_ breeding lines from two hybrid populations were included: (**A**) *LcFTa1* (Lcu.2RBY.6g043850); (**B**) *LcFTa2* (Lcu.2RBY.6g043870); (**C**) *LcFTb1-b2* (Lcu.2RBY.6g000730 and 6g000760); and (**D**) *LcFTc* (Lcu.2RBY.6g043940). On the left-hand side of each panel, parents ♀ Flip92-36L and ♂ ILL-1552 are shown, with three breeding lines designated 21/8 representing hybrid 1, while parents ♀ Krapinka and ♂ Flip96-48L with three other breeding lines designated 21/3, on the right-hand side, representing hybrid 2. Three developmental stages were used for sampling, as indicated in the Figure. Expression data were normalised using the internal reference gene, *LcElf4A-III* (Eukaryotic initiation factor 4A-III); presented as the average ± SE of four biological replicates (individual plants), used as non-interactive factors, and two technical replicates. Expressions for each parent were designated by different letters. Shared letters indicated the absence of difference within each hybrid population. Significant differences with confidence interval (*p* = 0.013 < 0.05; F = 3.273) are shown by different letters in a certain hybrid. Calculations were made using single-factor ANOVA with post-hoc Tukey test with residual diagnostics of initial data.

Diverse expression patterns were observed for the four *LcFT* genes (Figure 7). Parents, ILL-1552 and Krapinka, and corresponding breeding lines 21/8-3-F4-2 and 21/3-1-F4-3, with early flowering, showed much higher expression of the *LcFTa1* gene at the vegetative stage compared to the *LcELF3* genes, ranging between 5.20 and 3.28 expression units relative to the reference gene. Late-flowering genotypes had lower transcript levels, between 2.46 and 1.42 expression units at the initial time-points. All genotypes showed persistent *LcFTa1* expression before and during flowering, but mostly decreased levels in the post-flowering stage (Figure 7A). Notably, expression patterns of *LcFTa1* and *LcELF3a* genes were inversely related in most instances, with strong negative correlations before flowering, *r* = −0.732 (*p* = 0.016 < 0.05; *n* = 60) and during the flowering stage *r* = −0.812 (*p* = 0.0044 < 0.01; *n* = 60) (Figure 6A and Figure 7A).

The relative expression level of *LcFTa2* genes was very low, not exceeding even 0.25 expression units, the lowest of all genes examined, suggesting minimal responses (Figure 7B). The remaining genes, *LcFTb1-b2* and *LcFTc*, displayed similarly low levels and their responses appeared to be not significantly associated with flowering time; other functions are possible, perhaps in different tissues or ontogenesis stages (Figure 7C,D).

### 3.7. Seed Yield in Hybrid Breeding Lines and Germplasm Collection

**Figure 8:**
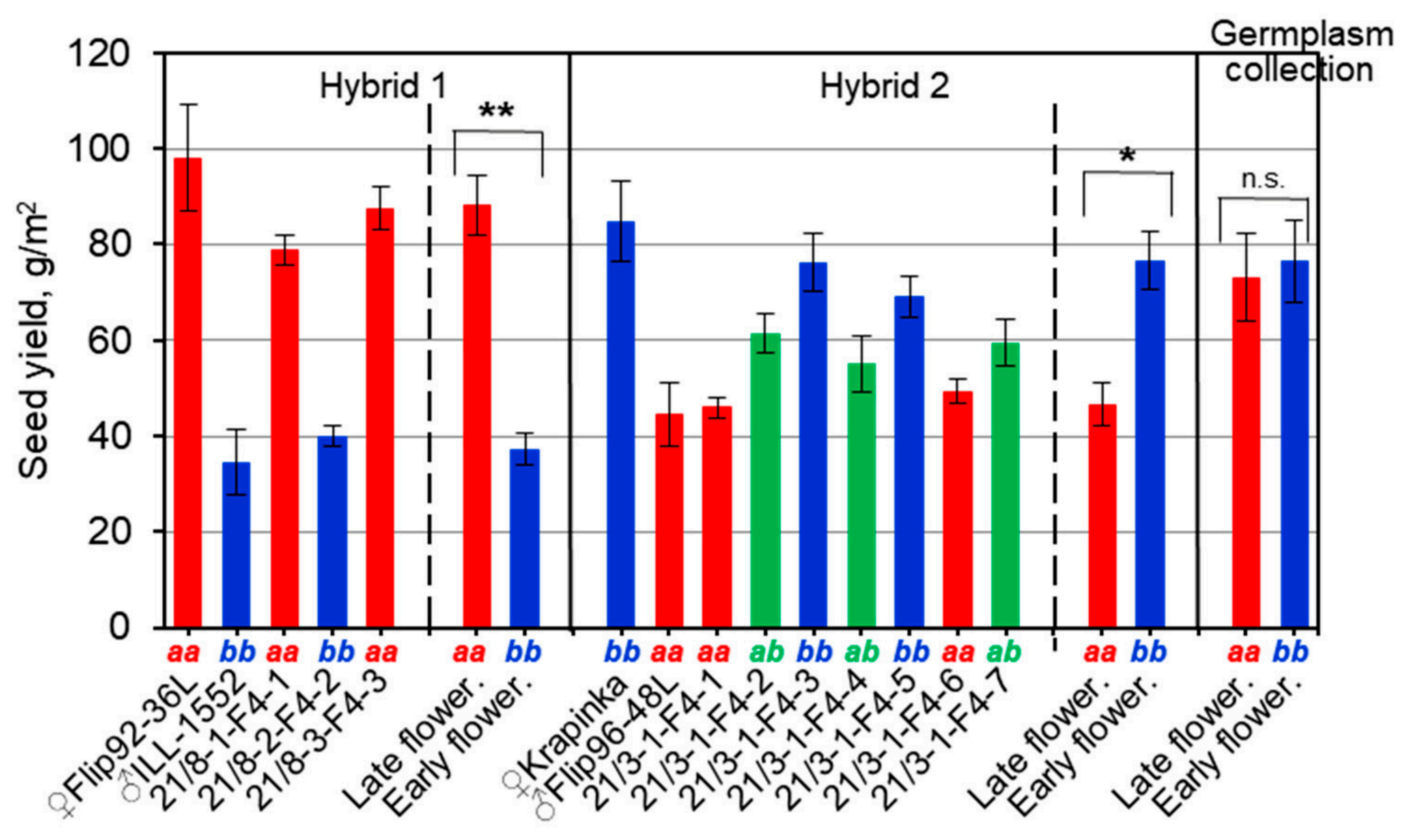
Seed yield study in two hybrids and the germplasm collection with late and early flowering. The data shown in red and blue bars represent late and early FT and corresponding genotypes *aa* and *bb* of the *LcELF3a* gene. Green bars represent heterozygotes *ab*. For the two hybrids, bars show the average for *n* = 10 plants ± SE. In the germplasm collection with late and early FT accessions, *n* = 37 and *n* = 27, respectively, results are shown as bars with average ± SE for three randomised plots during three-year field trials. Significant differences with confidence intervals (** *p* < 0.01; and * *p* < 0.05) are indicated for the comparison of pair groups within each of the two hybrids, and for the germplasm collection, using a *t*-test for two samples with equal variance. Field trials were carried out in KATRU research field, Akmola Region, Northern Kazakhstan, during three years. n.s., not significant.

The seed production of hybrid 1 and hybrid 2, comprising three and seven breeding lines, respectively, together with their parents, was assessed in terms of flowering time and genotyping of the *LcELF3a* gene. Additionally, average seed yield in the lentil germplasm collection of 62 accessions, including 35 late- and 27 early-flowering genotypes, were included in the evaluation (Figure 8). In hybrid 1, the maternal parent Flip92-36L with late FT and *aa* genotype, and two corresponding breeding lines, 21/8-1-F4-1 and -3, showed significantly greater seed yield compared to paternal parent ILL-1552 with early FT and another breeding line 21/8-1-F4-2 (*p* = 0.0032 < *p* = 0.01; *n* = 30) (Figure 8).

In contrast, in hybrid 2, the maternal parent, early-flowering cv. Krapinka, with the *bb* genotype, together with two corresponding breeding lines, 21/3-1-F4-3 and -5, had significantly greater seed yield compared to the paternal parent, Flip96-48L, with late FT and two corresponding lines 21/3-1-F4-1 and -6 (*p* = 0.0161 < *p* = 0.05; *n* = 36). Three heterozygous *ab* breeding lines, 21/3-1-F4-2, -4 and -7 were intermediate in terms of seed production. In the germplasm collection, average data for seed yield in the two groups of accessions with late and early FT, and corresponding genotype, were not different (Figure 8 and Supplementary Material S8).

In summary, these results indicate that the correlation between flowering time and seed yield was very weak; totalling the two hybrid populations together, *r* = 0.234 (*p* = 0.679), where parents with either late or early FT, and corresponding breeding lines, showed higher seed yield. It was also confirmed with no meaningful correlation, *r* = −0.058 (*p* = 0.641), in the lentil germplasm collection for two groups with early and late FT. Therefore, for the lentil genotypes and conditions used, flowering time can be combined with other traits for best selection of genotypes adapted to local conditions.

## 4. Discussion

Lentil exhibits substantial variation in flowering time (FT), much of which is associated with its geographic origin. The transition to flowering is controlled by a complex regulatory network in which the coordinated action of the flowering repressor *ELF3* and activator *FT* plays a central role [11,14]. In the current study, two distinct genes, *LcELF3a* and *LcELF3b*, were identified, consistent with previous reports [12,18]. Among these, *LcELF3a* was investigated as a key gene involved in the regulation of the FT trait in lentil [20].

Based on the sequencing results obtained in the current study, it was evident that seven lentil accessions examined, including commercial cultivars and parents of hybrid populations, did not carry the *elf3* mutation previously reported to cause a truncated LcELF3a polypeptide and a very early-flowering phenotype [23,24]. Therefore, the variation observed in FT among the studied germplasm collection and F_4_ hybrid lines was associated with natural genetic polymorphism in the absence of the *elf3* mutation. Similar findings have been reported in lentil germplasm collections from India [24] and Australia [21], suggesting that the *elf3* mutation is relatively rare and may be predominantly of Argentinian origin.

Nevertheless, despite the relatively small number of sequenced genotypes, the genetic polymorphism of the *LcELF3a* gene identified in the current study was quite extensive. Two haplotypes, A and B, characterised by six SNPs and one InDel in the second intron, were identified (Figure 3 and Table 1). None of the identified SNPs or InDels matched those reported previously [23,24], indicating a novel finding. More importantly, the haplotypes were significantly associated with the FT trait, and plants belonging to haplotype B exhibited early flowering at approximately the same time as cv. Precoz, which carries the *elf3* mutation (Figure 4). However, the identified SNP and InDel located in the intron region did not change encoded LcELF3a polypeptide, indicating genetic polymorphism in the corresponding *LcELF3a* gene. Therefore, the two haplotypes, A and B, were attributed primarily to differences in *LcELF3a* expression but not in the polypeptide structure. The difference in average FT between early- and late-flowering accessions was 4.4 days, which was still statistically significant in the current study (Figure 4). Similarly, a significant FT difference of only 1.5 days was reported between hybrid lines derived from a cross between cv. Redberry and accession ILL-1704 [19].

In the current study, the origins of accessions carrying haplotype B of the sequenced *LcELF3a* gene were highly diverse, including accession ILL-1552 from Iran and the cultivars Krapinka and Vekhovskaya from Kazakhstan and Russia, respectively. Therefore, the early-flowering habit associated with haplotype B is likely explained by factors other than the *elf3* mutation. These findings are consistent with previous reports showing that Australian accessions lacking the *elf3* mutation exhibited flowering times very similar to those of cv. Precoz [21]. Likewise, no association between the *elf3* mutation and early flowering time was reported in experiments involving lentil accessions in Canada [10].

The application of the developed ASQ marker LcELF3-SNP3 was demonstrated through the successful genotyping of the studied lentil germplasm collection, as well as hybrid breeding lines and their parental genotypes (Figure 5 and Table 2). The predictive accuracy of the used ASQ marker was very high and related to mismatches located only in boundaries between three groups of genotypes. Therefore, the genotyping with the tested ASQ marker was very successful, with a low level of possible limitation in the application in lentil molecular genetics and breeding. Similar genotyping studies of lentil collections have been reported previously; however, the authors employed different types of molecular markers, including SNP-derived allele-specific markers [24], KASP markers [21], and SNP markers identified through QTL-Seq analysis [18]. Additionally, the haplotypes identified in the current study were based on SNPs located in intron 2 of the *LcELF3a* gene. These findings are comparable to those reported recently in *Arabidopsis*, where one haplotype was also defined by an SNP located in the second intron of the *AtELF3* gene. Furthermore, the authors demonstrated that this SNP influenced the expression of the spliced transcript and affected plant responses to environmental growing conditions [28]. Similarly, the haplotypes identified in lentils in the present study were associated with contrasting FT phenotypes and were distinguished by SNPs located in intron 2, while the remaining regions of *LcELF3a* were monomorphic. Possible explanations for these observations include effects on splice regulation, enhancer activity, or chromatin structure; however, these hypotheses remain speculative and require further investigation.

All the results mentioned above emphasise the important role of the *LcELF3a* gene in lentil genotypes. However, the coordinated response of *LcELF3* and *LcFT* genes during plant transition to flowering remains unclear. It was inappropriate to use the germplasm in the current study to address this point due to the very diverse and non-adapted lentil genotypes included. In contrast, F_4_ hybrid breeding lines with their parents from two separate hybrids can show inheritance of the studied genes from either parent. Gene expression analyses of two *LcELF3* and four *LcFT* genes in selected F_4_ hybrid breeding lines and their parents in the current study were very challenging, but sufficient repeats and accurate analyses resulted in a solid conclusion about the coordinated interaction of *LcELF3* and *LcFT* genes, similar to that described recently [14]. In our study, two genes, *LcELF3a* and *LcFTa1*, had the highest expression with a complementary pattern indicating strong negative correlations before and during flowering stages, *r* = −0.732 and *r* = −0.812, respectively. This is consistent with gradually declining expression of the *LcELF3a* gene and activated expression of the *LcFTa1* gene during transition of lentil plants from the vegetative to the reproductive stage and flower initiation.

The strong negative correlations and leading role of *LcELF3a* and *LcFTa1* were confirmed in our study, where late FT plants from haplotype A had highly up-regulated repressor *LcELF3a* and lower mRNA production of activator *LcFTa1* at the vegetative stage, which was exactly the opposite trend observed in plants from haplotype B with early FT (Figure 6A and Figure 7A).

During the transition to flowering, the expression of *LcELF3a* was down-regulated but *LcFTa1* was increased in all lentil plants. It was followed by the shift in expression changes for early flowering in plants from haplotype B and with the delay in flowering in plants from haplotype A. This point showed biological variation and expression variability between lentils from haplotypes A and B in *LcELF3a* and *LcFTa1* expression with significant confidence intervals (*p* < 0.05) (Figure 6A and Figure 7A). The presented results were consistent with expression analyses of *LcELF3a* and *LcFTa1* genes reported earlier in lentil [20] and in barley [13] but differ from a recent study with QTL analysis in a lentil RIL population from the cross ILL-8006×cv.Milestone [22]. The role of *LcELF3b* might be to make a smaller contribution to the transition of lentil plants to flowering due to the four times lower transcript level, and possibly is expressed in other plant tissues or at different time-points. These results were similar to those described in pea, which found the *ELF3b* gene had limited effect on FT but was related to photoperiod sensitivity [12].

Very low, almost negligible, expression levels of *LcFTa2* were observed in the current study, which contrasts with previously published results [20]. The reasons for these differences remain unclear but may be related to variations in the origin of the lentil genotypes, the tissues analysed, or the developmental stages sampled. This issue warrants further investigation. Slightly higher expression levels were recorded for *LcFTb1-b2* (analysed as a combined gene group) and *LcFTc*; however, the mRNA production levels of these genes were not associated with the FT trait in the F_4_ breeding lines and their parents. The obtained findings suggest that these genes may play roles other than the direct regulation of flowering time in lentil. The expression pattern of *LcFTc* was consistent with previous reports [20], whereas different results were obtained for *LcFTb1* and *LcFTb2*, which have been reported to be expressed and associated with an early-flowering phenotype in lentil plants [21,22].

The final set of results from the current study showed no significant differences in seed yield between lentil accessions with early and late FT in the germplasm collection. In contrast, highly variable results were observed among the two hybrid populations, where genotypes with either early or late FT exhibited higher seed yield depending on the genetic background (Figure 7). As discussed above, previous studies in lentil have reported contrasting findings regarding the relationship between FT and seed yield, and some studies have found no association between these traits [36], which is consistent with the results of the current study. Despite the importance of flowering time, the present findings indicate that FT alone is not a reliable predictor of seed yield, even among lentil genotypes with defined molecular profiles. The results further suggest that seed yield may be more strongly influenced by the overall adaptation of a genotype to local growing conditions, regardless of whether it exhibits early or late flowering. Therefore, FT should be considered together with other agronomic traits when evaluating and selecting superior genotypes for specific environments.

## 5. Conclusions

(1) Sequencing of the *LcELF3a* gene in the lentil accessions studied confirmed the absence of the *elf3* mutation which is known to cause a truncated polypeptide and very early-flowering phenotype in lentil plants.

(2) Six SNPs and one InDel representing natural genetic polymorphism in the absence of the *elf3* mutation were identified and the studied genotypes were classified into two haplotypes, A and B, which were significantly associated with late and early FT, respectively.

(3) Genotyping using the ASQ marker LcELF3a-SNP3 revealed clear allele discrimination in both the lentil germplasm collection and the hybrid breeding lines. The homozygous genotypes *aa* and *bb* identified by LcELF3a-SNP3 showed significant associations with late and early FT, respectively.

(4) Gene expression analysis of F_4_ hybrid breeding lines and their parents revealed high mRNA levels of two coordinated genes: the flowering repressor, *LcELF3a*, and the flowering activator, *LcFTa1*. The expression profiles of these genes were negatively correlated during the vegetative and flowering stages (*r* = –0.732 and *r* = –0.812, respectively). Expression of the *LcELF3a* gene gradually declined, whereas flowering initiation was associated with the up-regulation of *LcFTa1* expression.

(5) Expression of the *LcELF3b* gene showed a pattern similar to that of *LcELF3a*, although expression levels were approximately four times lower. None of the other three *LcFT* genes (*LcFTa2*, *LcFTb1-b2*, and *LcFTc*) showed significant effects on, or associations with, the FT trait in the studied hybrid breeding lines and their parents.

(6) Seed yield was significantly higher in early FT cv.Krapinka and in late FT parent Flip92-36L, and in corresponding breeding lines, with *aa* and *bb* genotype of *LcELF3a*, respectively. No correlation, *r* = −0.058 (*p* = 0.641), was found between early- and late-flowering genotypes in the lentil germplasm collection. Therefore, FT should be considered together with other agronomic traits when evaluating and selecting the most suitable genotypes for specific local environments.

## Figures and Tables

**Table 1:** Haplotype identification of the *LcELF3a* gene among seven lentil accessions grouped for time to flowering, with six SNPs and one InDel profile. Nucleotides in SNP and InDel positions are shown in bold and are accompanied by the two neighbouring nucleotides from both sides. Numbers in brackets indicate SNP and InDel positions in the annotated sequence accession JX946295, according to data published earlier [24].

Haplotype	Genotype	Time of Flowering	SNP and InDel Positions from ‘Start’ Codon of *LcELF3a* Gene, bp						
			1435 (1469)	1440 (1474)	1524 (1558)	1710 (1744)	1757 (1791)	1869 (1903)	1974 (2008)
A	Flip92-36L, Flip96-48L, Niva-95, Syrian Local	Late	GG**T**GG	TT**T**AT	AA**A**TT	AA**G**AA	TA**G**TT	AG**-**CC	TG**T**AC
B	ILL-1552, Krapinka, Vekhovskaya	Early	GG**C**GG	TT**A**AT	AA**T**TT	AA**A**AA	TA**T**TT	AG**A**CC	TG**C**AC

**Table 2:** Comparison between genotyping with LcELF3a-SNP3 molecular marker scores and phenotyping of plants for FT trait in the lentil germplasm collection. Kruskal–Wallis test was used to estimate statistical differences between genotype groups with corresponding phenotypes. H_stat_ means results of test statistics; d.f, degrees of freedom.

Genotype	Fluorophore	Number of Genotyped Accessions	Number of Phenotyped Accessions	Number of Mismatches	Average FT, Days	Kruskal–Wallis Test
** ***aa*** **	FAM	31	37	6	41.5 ± 0.3	H_stat_ = 54.163, d.f. = 2, *p* = 1.73 e^−12^, *p* < 0.0001
***ab***	FAM/HEX	9	2	7	39.7 ± 0.0	
***bb***	HEX	26	27	1	38.1 ± 0.4	
***Total***		66	66	10	40.0 ± 0.3	

**Note:** Genotypes, ***aa***, ***ab*** and ***bb***, were designated by italics and bold, with red, green and blue colour, respectively, similar to those present in Figure 5 above. Fluorophores, FAM and HEX, were also designated with red and blue colour, and both fluorophores are present in heterozygous genotypes ***ab***, similar to Figure 5 above.

## Data Availability

The original contributions presented in this study are included in the research paper and the Supplementary Material. Further inquiries can be directed to the corresponding authors.

## Supplementary Materials

The following supporting information can be downloaded at: https://www.mdpi.com/article/10.3390/plants15203147/s1. Supplementary Material S1: List of 66 lentil accessions used in this study. Supplementary Material S2: Sequences of encoded ELF3 proteins in lentil and other studied legumes used for molecular phylogenetic analysis. Supplementary Material S3: Sequences of primers and entire *LcELF3a* (Lcu.2RBY.3g063730) gene in lentil used in this study. Supplementary Material S4: Details of allele-specific primers and universal molecular probes used for ASQ genotyping. Supplementary Material S5: Details of primer sequences with amplicon sizes used for qPCR. Supplementary Material S6: Sequence alignment of fragments in *LcELF3a* gene with identified SNPs in two haplotypes, A and B. Supplementary Material S7: Histogram of 66 studied lentil accessions for days to flowering for three studied years. Supplementary Material S8: Genotyping of lentil germplasm collection for *LcELF3a* gene and phenotyping results for flowering time and seed yield traits. Supplementary Material S9: Results of Kruskal–Wallis test for the comparison between genotyping of three groups of genotypes (*aa*, *ab* and *bb*) with corresponding phenotyping results of lentil accessions for FT presented in Table 2.
