# Supplementary material for: Genotyping and Expression Analysis of the Non-Truncated LcELF3 Gene Shows a Link with Early Flowering in Lentil (Lens culinaris Medik.)
Source: Plants (Basel). 2026 Oct 15;15(20):3147. doi: 10.3390/plants15203147 (PMC13709218; doi:10.3390/plants15203147)
Supplement: Supplementary file 1 [file plants-15-03147-s001.zip › Supplementary-material S2-S9.pdf]

Supplementary material S2. Sequences of encoded ELF3 proteins in lentil and other studied legumes used for molecular phylogenetic analysis

ELF3a

***Lens cuinaris* Medik.**
>LcELF3a1-2RBY.3g063730-Redberry
MKRGSDDEKMMGPLFPRLHVGDTEKGGPRAPPRNKMALYEQFSIPSQRFN
LPLHPNNSTNSVPPASSSQGTVHERNYIFPGHLTPETLIRQAGKHLSRQS
KGANLNGSIAQIEHRKKVDEDDFRVPVYVRSNIGQSNEKRPESFDGKRLP
STGSRYFGFLKPGKIDRERELIQNGSTVVNAGTDVRNEIDGPPQVSPNKE
HPVTSARNASTGERVDALVRQVKVTPNQEVQDRRVFKHSSLRQGDARLRQ
DCRAESQSNGHGQSDGLLESTREVDTSNGPIVNHISPTQAINDTEYHDTG
TGSPKQLGNLNKNDNISKISRVENLSTVKISPDDVVAVIGQKHFWKARKA
IANQQRVFAVQVFELHRLIKVQQLIAGSPDLLFDDGAFLGKSLPDGSTPK
KLPLEYVVKTRLQNLKRKVDSEKINQNMECSAENAVGKTSISSVKNTSHL
SSSMPFAGNPHQGNMAADNGMGPWCFNQSPGHQWLIPVMSPSEGLVYKPY
PGPGFTGTNFGGCGPYGASPSGGTFMNPSYGIPPPPEIPPGSHAYFPPYG
GMPVMKAAASESAVEHVNQFSAHGQNHRLSEDEDNCNKHNQSSCNLPAQR
NEDTSHVMYHQRSKEFDLQMSTASSPSEMAQEMSTGQVAEGRDVLPLFPM
VSAEPESVPHSLETGQQTRVIKVVPHNRRSATESAARIFQSIQEERKQYD
AP

***Lens cuinaris* Medik.**
>LcELF3a2-2RBY.5g070480-Redberry
MSGGKVDAEKVGPMFPRLHVNDTAEKGGPRAPPRNKMALYEQFSVPFQRF
NPNSNSNSNSSYSLPLTPSSTLGNDPERSYIFPVCLPSQTENYISHQSNG
AHTLSAQIEHKKKVDGDDSRAYDCSRIGKSNDKTMKSFNGEKHTPTGSGN
FGCLVSGKNDGDKDQTQFGSLVVEMRKDVRNKGEAHLQESLSRQKTVISV
ERISTGENIDSPVRQAEMISDEEDQSDACVQQESNNIEHGGSLVDSAMDM
DNRNSLVLRGCFRSSVNQTNVPETANHTEYHDTNIDSPIENGNSEGSDDL
SKNSTLENMPSPKLSPDGVVEILGQQLFWKARRKITNQQRMYAVQVFELH
RLIKVQHLIAESSNLLLDSAAILGKSPLRGSNSKSLSLEEVDEPQAQNHK
QQDHSENRNHELDCSPVNGVEKTSFSSQKYGSHLSNYTPFSGNSDQANFG
AHCFNQPPGHQWLVPVMSPSEGLVYKPYPGPAFPGSVYGGYEPFGHSPAD
GTFMNPAYGVPNFHQAIAMSPFIPPSSYAYFPPHGVPAMNQSTSRSAAEQ
VNQFVAHGSRDRNNNSSLVGAGFDTHNQSSCDFSNQKSGPILHVTKARPS
RERELASSPNEKAQGIRIEKSSEGRDAFPSSFTVPLVSDEVFQPLETRQK
PQVIRVVPHNPRSATVSAARIFQSIQEERKQYALF

***Pisum sativum* L.**
>PsELF3a1-Psat5g037560-Cameor
MLVIRRKEGLEHHLGIKWLCMSNLVFPLKGLTCHSTRILRTIRFPPASSS
QGTVHERNYVFPGHLTPETLIRQAGKHLSRQTKGANLNASLAQIEHRKKV
DEDDFRVPVYVRSNIGQSNEKRIESFDGKRPPSTGSRYFGFLKPGKSDRE

RDPRQHGSAVVNAVTDVRNDIDGPPQVSPNKEHPSSSARDGSIGEHSDTL
VRQGKVTANQEVQDRRVFKLSSLRQGDARLRQDCRAESQSNGHGQSDSLL
ESTREVDKSNGPIVNQTSPTQAINGTEYHDTGTGSPKQLGNSNKNDSISK
ISRVEDLSTVKISPDDVVAVIGQKHFWKARKEIANQQRVFAVQVFELHRL
IKVQQLIAGSPDLLFDDGAFLGKSLPDGSTPKKLSLEYVVKARLQNLKRK
VDSEKINQNMECSAENAVGKTSISSVKNTSHLSSSMPFAGNPHQGNVAAD
NGMGPWCFNQSPGHQWLIPVMSPSEGLVYKPYPGPGFTGTNFGGCGPYAA
APSGGTFMNPSYGIPPPPETPPGSQAYFPPYGGMPVMKAAASESAVEHVN
QFSARGQSRRLSEDEADCNKHNQSSYDLPVQRNGATSHVMYHQRSKEFEV
QMSTASSPSEMAQEMSTGQVAEGRDVLPLFPMVPVEPESVPHSLETGQKT
RVIKVVPHNRRSATESAARIFQSIQEERKQYDAL

***Pisum sativum* L.**
>PsELF3a2-Psat0s304g0040-Cameor
MSRGKGDAEKVGPMFPRLHVNDTAEKGGPRAPPRNKMALYEQFSVPFQRF
NPNSNSNSSNSLPLTPSSNLGNDPERSYISHQSNGANTHTLSAQLEHRKK
VDGDDSRAYDHSRIGQSNDKTMKSFNGEIITPTGSRNFGCLVSGKNDSDK
DQTQFGSLLVEMRKDVRNKGEVHLQESLSRQKTVMSVESILTGEIIDSSV
RQAEKISDEEDQNDACLQQESNDIEHGGSLVDSAMDMDNRNSLVLRGCFR
STVNQISVPEAADHTEYHDTNIDSPIENGSSEGSDDLSKNSTLENMPSPR
LSPDGVAEILGQQLFWKARRKITNQQRMYAVQVFELHRLIKVQHLIAESS
NLLPDTAAVLGKPLLQGSNSKSLSFEEVVEPQAQNHKQQDHSENQNHKLD
YSTENGVGKTSLSSQKSNQANAGSQCFNQSPGHQWLIPVMSPSEGLIYKP
YPGPGFPGAVYGGYEPFGHSPPDGTFMNPAYGVPNFHQAIAMSPFIPPSS
YAYFPPQGVPAMNQSASGSVAEQKSGAVLHVTKSRPSRERELASSPNEKA
QGIRIEKSSEGRDALLSSFTVPLASDEVFQSLETRQKPQVIRVVPHNPRS
ATVSAARIFQSIQEERKQYDLF

***Cicer arietinum* L.**
>CaELF3a-Ca14853-Frontier
MKRGKDDEKMMMGPLFPRLHVGDTEKGGPRAPPRNKMALYEQFSIPSQRF
NLPLHPNTSTNTVPPSSSIQGTVLERNYVFRGHLTSQTPIHQAEKRSSLQ
LEGVNLNTSLSQLEQRKKVDEDDFMVPVYVRSKIGQSNDKSLESFDGKKL
NSTRSRYFGFSKAGKTDCERDPKQYGSHLVNTKIDVRNEIDGPPQVSPNK
EHPFTSVRDISTGESVDTLVRQAKVTLNQEFSDCAVFKFSSLRQVDACSR
EECGTESQSNGIRQSNTLVESTRGVDKSNDPIANQTSPTEAINGTEYKDT
WTGSPIQKGNLNRKDNISKISGVENLSTLKISPDDVVAIIGQKHFWRARK
AIANQQRVFAVQVFELHRLIKVQQLIAGSPDLLFEDGAYLGKSPPVGCTT
KKLSLEYVVKPREQNLKRKDDSEKINQEMECSAENAVGKTSISSVKNGSY
LSTATPFAGNPHQGNMAADSGMGPWCFNQSPGHQWLIPVMSPSEGLVYKP
YPGPGFTGTNCGEYGPIGAAPFMNPSYGMPAPPETPPGSHAYFPPYGGMP
VTKAAVAESAVGHVNQFSAHGQNDHLSEVEANHNKHNQIPCNLPAQRNGA
TSHVMNRQRHKEFELQGSTASSPSEMAQGMSIGQVSEGRDVLPLFPMVPL
EPEVVPQSPETRQQSRVIKVVPHNRRTATESAARIFQSIQEERKQYESL

***Vigna angularis* (Willd.) Ohwi & H.Ohashi**
>VaELF3a1-Va07G066410-Jingnong6
MKRGNDDEKVAGPMFPRLHVNDAEKGGPRAPPRNKMALYEQLSVPSQRFN
PRLLPLKPNSSSNLAAPPTSSTQGSEHGRSYGYPVRLPSQTPAHRAESYI
SRQSDGSRSNTSSVQLERRKRADEDDVHAYICSRIGHSNDKAINRKKFTP
LGGSNFRSVSVQNDGERDPAQFGSLPVDMRKHVRRGNETHPHVSSSRQQP
KMSVKNKSSGEVIDSLVMQAKVIPNLEDQDCSVPSIGRLHQDGACLQQEC
GAGPQSGDVDLLKSTRDMDNGNALVPKSCFHSAVNQTCPVEVTDDVDVEY
HDVGTEGPLQKGNFDESGDVSKISMVTNLSSLIVSPDDVVGVLGQKHFWK
ARRKIANQQRVFAVQVFELHRLIKVQQLIASSPDVLLEDGAFLGKFPLEG
STPKTISLQVVVDPKQQTPKQKNNSEKQNLKTECSAENAVEKKTSFSSPK
NGSHITNHTPFSGIPHQADVASDNRTSPWSFNQSPQHQWLIPVMSPSEGL
VYKPYPGPAFTRTNPAGCGGPFGQAPLGATFMHPASQFPASHQVVGVSPF
VSLPNHTYFSPSGMPVVNQAASGSAVEHVNQFAGQGSHGQNGHSSVEGAN
FNTQHNQSSSNLPVQRNGAMSHVKKLRVSKERRLQGSTASTPTETPQGIR
AGSIAEGSDAHSLSFHSAETRQQTQVIKVVPHNGKSATESAARIFQSIQE
ERKQHDLV

***Vigna angularis* (Willd.) Ohwi & H.Ohashi**
>VaELF3a2-Va04G066540-Jingnong6
MKRRKDDEKVMGPMFPRLHVNDTEKGGPRAPPRNKMALYEQFSIPSQRFN
SGVVPLNQNISSNTAPPTSSSQGTVPERNNVFPVHLPSQKPIRPVEKCHS
RQSEEANLTCSLEQRKKVYEEDFRVPVYVHSRVGQCNDKSVETFDRKKIT
HTGTRYFGCSVAGQSDCERVPKQFGSSHVRKDARCETDGLPQVSTSKDQP
LTSVRSISTRENIDTLVRQAKVTPNQEFQDCHVSKRNRFRQDDGYLRQDC
GVGSQSNDIGHSGSLVQSSRKLGNGNAATANQTNLAEAINDTGHHDTGMG
SLIRGGKLNGSDNASKISSVDNLSPVNISPDDVVGIIGQKQFWKARRAIS
QAVSSLNIILCWRAPLYSISSHDSLSSSHWILDMFLSSFPLAWFSMSPLG
QVSLHYTEGLELRRGTNQQRVFAVQVFELHRLIKVQQLIAGSPEVLLEDG
TFLGKSTPEGSTRKKLSLEYVVKPWQQNLMRKDDSEKLNHKMECSAENAV
GKTSLSSVKNDSHLSNYTPFSRNPQQANVAADSGMGPWCFHQSAGHQWLV
PVMTPYDGLVYKPYPRPGFTETDGGGCGPSPFGGNFMNPAYGIPNSLQGV
GVSPQTPPGSLAYFPPYGMTVMNATISESAVDQVNQFSSLGSHRYNGHLP
GGEADHITNNQSSFNLPTPRKGAFSHVLKYQTSKDFELQGSTASSPGEMA
QGLSTGQVAEGRDVLPLFPMVAAEPESIPRSLETGQPTRVIKVVPHNRIS
ATASAARIFQSIQEERKQYDSV

***Phaseolus vulgaris* L.**
>PvELF3a-001G032900-G19833
MKRGNEDEKVAGPMFPRLHVNDAEKGGPRAPPRNKMALYEQLSVPSQRFN
PRLLPLKPNSSSNLVAPPVSSTQGNGHERSYAYPVRLPSQIPAHRAESYI
SRQSDGSRSNTSSVQLERRKRADEDDVHAYICSRIGHSNDKTMKSFNGEK
ITPLGARNFCSVSVQDDGERGPTLFGSLPVDMRKHVRSGNEAHPHVSSSR
QQQKMSVKNKSSGEVIDSLVMQAKVIPNQEDQDCSVPSLSRSHQDGACLQ
QECVAGPQSNDVEHRNGLLSSTRDMDNGNALVPKSCFYSAANQTCPVEAT
GDVEYHDIGTEGPMQKGIFDEIGDVSKISTVTNLSSQVVSPDDVVGILGQ
KRFWKARRKIANQQRVFAVQVFELHRLIKVQQLIASSPNVLIEDGAFLGK

FPLEGSTPKTISLQVVVEPQQQNPKRKNNSEKPNHKTECSAENAVEKKTS
FSSPKNGSHLTNHSPFSGIPHQTDVASDNGTSPWSFNQSPQHQWLIPIMS
PSEGLIYKPYPRPAFTGMMPAGCGGPLGQAPLGATFMNPAYQFPASHQVV
GVSPFVSPANHTYFSPYGMPVVNQAASGSAAEHVNQFATQGSHGQNGHSS
VEGANFNTHHNQSSSNLPVQRSGAMSHVKKLQVSKERQLQGSTASTASET
PQRIKARNITEGSDAHSLSLHSVDARQQTQVIKVVPHNGKSATESAARIF
QSIQEERKQHDLV

Vigna unguiculata (cowpea)
>VuELF3a1-Vigun09g227400-IT97K-499-35
MKRGKDDEKVMGPMFPRLHVNDTEKGGPRAPPRNKMALYEQFSIPSQRFN
SGVLPLNPNISSNTAPPASSSQGTVPERNHVFPVHLPSQKPIRPVEKCHS
RQSEGANLTSSLEQRKKVYEEDFRVPVYVHSRVSHGQCNDKSVESFDRKK
ITHTGTRYFGCSVAGQSECERVPKQFASSHVRKDARGETDGLPQVSTSKD
QPLMSVRSISTRENIDTIVRQAKVTPNQEFQDCHVSKVNRFRQGDACLRQ
DCGVGSQSNDIGHSGSLVQSSRKIGNGNAATANQTNPAEAINDTGHHDAR
TGSLIQGRKLNGSDNASKISSVDNLSTLKISPDDVVGIIGQKQFWKARRA
IAHQQRVFAVQVFELHRLIKVQQLIAGSPEVLLEDGTFLGKSPPKGSTRK
KLSLEYVVKPWQQNLKRKDDSEKLNHKMECSAENAVGKTSLSSVKNDSHL
SNYTPFPRNPQQANVGADSGMGPWCFHQSAGHQWLVPVMTPSEGLVYKPY
PRPGFTGTDGGGCGPAPFGGNFMNPAYAIPTSHQGFGVSPQTPPGSLAYF
PPYGMTVMNATMSESAVDQGNQFSSLGSHRYNNGHLPGGEADHITNNQSS
CNLPTPTNGALSHVLKYQTSKDFELQGSTASSPGEMGLGLSTGQVAEGRD
VLPLFPMVPAEPESIPRSLETGQPTRVIKVVPHNRISATASAARIFQSIQ
EERKQYDSV

***Vigna unguiculata* (L.) Walp.**
>VuELF3a2-Vigun08g035200-IT97K-499-35
MKRGNDDEKVAGPMFPRLHVNDAEKGGPRAPPRNKMALYEQLSVPSQRFN
PRLLPLKPNSSSNLVAPPTSSTQGSGHERSYGYPVRLPSQTQAHRTESYV
SRQSDGSRSNTSSVQLERRKRADEDDVHAYICSRIGHSNDKAMKSFNGKK
FTPLGNFCSVSVQNDGDRDSAQFGSLPVDTRKHVRSGNETHPHVSSSRQQ
QKMSVKNKSSGEVIDSLVMQAKVIPNQEDQDCSVPSISRLHQDVACLQQE
CVAGTQSNDVEHRNGVLNSTRDMDNGNALVPKSCFHSAVNQTCPVEVTND
VDVEYHDVGTEGPIQKGNFDESGDVSKISTVTNLSSLIVSPDDVVGILGQ
KHFWKARRKIANQQRVFAVQVFELHRLIKVQQLIASSPDVLLEDAAFLGK
FPLEGSTPKTISLQVVVDPQQQTPKRKNNSEKQNLKTECSAENAVEKKTS
FSSPKNGSHLTNHTPYSGLPHQADVASDNRTSPWSFNQSPQHQWLIPIMS
PSEGLVYKPYPGPAFTGAMPAGFGQAPLGATFMNPAFQFPASHQVVGVSP
FVSPANHTYFSPYGMPVANQAASGSAVEHVNQLAGQGSHGQNGHSSVEGA
NFNTHHNQSSSNLPVQRNGAISHVKKLHVSKERQLQGSTASTPGETPQGI
RAGNIAEGSDAHSLSLHSVETRHQTQVIKVVPHNGKSATESAARIFQSIQ
QERKQHDLV

***Vicia faba* L.**
>VfELF3a1-Vfaba.2g139200-Tiffany
MKRGNDDEKMVGPLFPRLHVGDTEKGGPRAPPRNKMALYEQFSIPSQRFN
LPLHPNTSGNSVPPPASSSQGTVHERNYVFPGHLTPETLIRQAGKHLSRQ
LEHRKKVDEDDFRVPVYVRSNIGQSNEKRPESFDGKRPFPTGSRYSGFLK
PGKVDWERELKQHGSAVVNAGTDVRNEIDGPPQVSPNKEHPFSSARDEST
GERIDTLLRQAKVTPNQEVQDCRVFKHSSLHQGDARLRQDCRAESQSNGH
GQSDGLLESTREVDKSDGPIVNQTRLTEAIDDTEYHDTGTGSLKQLGNLN
KNDNISKISRVEDLSTVKIFPDDVVAVIGQKHFWKARKEIANQQRVFAVQ
VFELHRLIKVQQLIAGSPDLLFDDGAFLGKSLPDGSTPKKLSLEYVVKTQ
LQNLKRKVDSEKINQNMECSAENAVGKTSISSVKNTSHLSNSMPFAGNPH
QGNMAADSGMGPWCYNQSPGHQWLIPVMSPSEGLVYKPYPGPGFTGTNFG
GCGPPFGAAPSGGTFMNPSYGIPPPPETPPGSHPYFPPYGSMPVMKAAGS
ESAVEHVNQFSARGQNRRLSEDEADCNKHNQNSCNLPVQRNGTTSHVMYH
QRSKDFELQMSTASSPGEMAQEISTAQVAEERDVLPLFPMVPIEPESAHS
LETGQQTRVIKVVPHSLETGQQTRVIKVVPHNRRSATESAARIFQSIQEE
RKQYDGL

***Vicia faba* L.**
>VfELF3a2-Vfaba.3g177360-Tiffany
MSRGNGDAEKVGPMFPRLHVNDTAEKGGPRAPPRNKMALYEQFSVPFQRF
NPNSISLPQTRSSSLGNDPERSYIFPVCLSSQTESYISHQSNVANMDALS
ALEHRKKVDGDDSRAYDRKRIGQSNDKTMKSFNGETLTPTGFGNFGCLVS
GNVGDKDQTQFGSLLVKMRKDVRNKGEAHLQESLSTQKTVMPIERLSTGE
IIDSPVRQAEKISDEEDQRDACLQQESNYVEHGGSLVDSAMDMDNRNSLV
LRGCFRSTVDQTSVLEAANHTEYHDTNIDSPIDNGTSEGSDDVSKNSTLE
NMPSPILSPDGVVEILGQQHFWKARRKITNQQRMYAVQVFELHRLIKVQH
LIAESSNLLLDAAAILGKSPLQGSNSKSLSLEEVVEPQAQNHKQQDHSEN
QNHKLDCSAENGVEKTSFSSQKYGSHLSNYTSFPGNSDKTNVGAQCFNQS
PGHQWLIPVMSPSEGLVYKPYPGPGFPGAVYGPPDGTFMNPAYGVPNFHQ
AIAMSPFIPPSSYAYFPPPGMPAMNQSVSGSVAEQVNQFVAQGSLERNSN
SSLVGADFDTHNQSSRNFSNQNSGAISHVTKSRPSRERELACSPNEEAQG
IRIEKSSEGRDARPSSFTVPLVSDEVFQSVETRQKPQVIRVVPHNNPQSA
TVSAARIFQSIQEERKQYDLL

***Lupinus angustifolius* L.**
>LaELF3a1-R30650-Tanjil
MKKGKDDEKIVGPMFPRLHVNDKEDKGGPRAPPRNKMALYEQLSIPSQRF
NHGVLLQNPDTSSNNVPPTSTSQGAGLERNYGFPAYLPSQTSTRRAEKYT
SRSYGANLNASPVQFQKRKKVDEDDLRVPVYIHSRTGQSNSKNLESFDWK
KLTLAPKGCRYIGCSTAGQNDFERDPKQFGSLFVNMRKDVRSETKGLVQV
TPSKEQPIKSVRDLSSTGENVDTLVRQAKVTPNQEFRDSPASKHNMLHQG
DDTFLQREREYGSQFNDTRQGDGLVEPTRETDNGNAAVSNQTTPTVAIND
TEYHVIRAGKLESVSTLKVSPDDVVEIIGQKRFWKARKAIASQQRLFAVQ
VFELHRLIKVQKLIAGSPDLLLEHAAFLGKSPLKGSTPKKPALEFVVKPQ
PQTLKRKHESEKANHKMECSAENAVGKTSLSPMKNGSHHSNYTPFSRNQH
TANVPADSGMPPWCFQQSPGHQWLIPIMSPSEGLVYKPYPGPGFTGTTCE

GGYGPTPLGGTFMNAAYVIPASHQENGVPKNTPPSSHAYYPPYRMPVVNA
AMSESAVEQVKQFSEHASHGQNGHLNGRETDINTNNQSSCDLPVQRNRAI
SHAMKKYQTSKEVELQGSTASSPSEMAQGISTRQIAEGRDALPLFPMAPV
AAPEEVPQSLETGQQPRAIKVVPHNPRSASESAARIFQSIQNERKQHDLV

***Lupinus angustifolius* L.**
>LaELF3a2-R00361-Tanjil
MKREKDEEKVVGPMFPRLHVNDTEKGGPRAPPRNKMALYEQFSIPSQNLN
QLGSSSQGTGSGRSYIIPCHLPSQMPTHRAEIYISHQSNRARQNTSSAHL
EQRKKVDEDDFMVPVYVDSRTGQCNDKRLESFDRKKLTSMGSRNFGCSLE
VQKDGGRHLKQLGSPLTNMREDASSESEGFAQVSPSRDPPAKNISTVETT
KSLVNQTKVIQYQDCPASFVSSLCQADDCLQQECVAGSQSNNIGHRHREV
LLDSIGDTDKGNTTLPMGCFRSPTNQTSPVEAMHETEFDDTTRTASPVQK
GSSDESDDVSKISSIGNLSSLKASPDDVVGVLGQKHFWKARRKIANQQRV
FAVQVFELHRLLKVQHLIARSPDLLLEDGAFMGNSPPKRSTPNLTLEVVV
KPQLQNHKRKDDSEKLNHNKMECSAENAVGKRSCSTPKNGSHHSNYTPFA
GNLHQTEAAADNRMGSWGFNQSPGHQWLIPVMTPSEGLVYKPYPGPAFPG
TMCGGGCGPFGPGPPGGTFMNPSYGVPPHISPGSLTYFPPYGMPVMNQAV
SGSADEQVNHFAAQGSHDQNGHSLVEGANFNTHNQVSNNSPVQRKGAISH
VKKSHASKKSELQGSTASSPSEKAQGIRKRQIADGRDAHSLSHVAALIPE
GDLQSLETRPQTRVIKVVPHNRRSATESAARIFQSIQEERKQYDLM

***Cajanus cajan* (L.) Huth**
>CcELF3a1-KK1_023036-Asha
MKRGSEDDKVVGPMFPRLHVNDAEKGGPRAPPRNKMALYEQFSIPSQRFN
PRLLPLNPNPSSNKVSSSTPSTQGNGHERSHVYPVCLPSQTPSHRAESYI
SRQSDGARSNTSSVQLEQRKNVDEDDIHAYIRSRVGQPSDKTLESYKGKK
FTTLGSRNVCFSVAVQNDGDKDLTQFGCVPVDMRKDVRSGNEVHPQVSSS
RQQRKITVRNKSSGEIIYSPVMQVKVIPNQEDQDYSVPNISRLHQDNAPP
QQKCVAGSQSNDIEHCDVLLNSTRERDVDNGNALVPKGCFHSAVNQTRLL
EATIDAEYHDAGTGGPIQKGNFDESGDVSKISTVTNLSSLIVSPDDVVGI
IGQKHFWKARRQIANQQRVFAVQVFELHRLIKVQQLIAASPDVLLEDGAF
VENFPVPGSTPKNLSLEVIVEPQQQNHKRKNDSEKPNHKTECSAENAVSR
KTSFSSPKNGSHLTNHTPFSGTPHQANVTSDNRTSPWCFNQSPGHQWLIP
IMSPSEGLVYKPYPGPEFTGTMHGGCSVPFGQAPIGTTFMNPAYQFPASH
QVVGVSPFVPPASHAYFPPYGMPVMNQAASGSAVEPVNQFAAQGSHGQNG
HSSVEGANFNTHPNQSSSNLPVQRNGATLHVKKSQASKERQLQGSTTSSP
SEMAQGISAGKIAEGSDAQSLPLPSVATRQQTQVIKVVPHNRKSATESAA
RIFQSIQEERKQHV

***Cajanus cajan* (L.) Huth**
>CcELF3a2-KK1_009573-Asha
MPQVLIGRVLCKFLDPTPVPLYSQQRVFAVQVFELHRLIKVQQLIAGSPD
ILVEDGTFLGKSPAKESTPKKLPLEYVVKPRQQNLKHKDDSEKLNHKMEC
CAENAVGKTSLSSVKNGSHLSNYTPFPGNPQQTNVGADSGMGPWCFHQSP
GDQWLIPVMTPSEGLVYKPYPGPGFTGTGYGGCGPAPLGGAFMNPAYGIQ
TSHQGDTPPGSHGYFPPYGMPFMNAAMSESAVEQVNQFSALGSRGHLSRG

EGNLNTNNQSSCNLPVQKNGAMSHALKYQTSKDFDLQGSTASSPGEMAQG
LSTGQHAEGRDVLPLFPMVSAEPESAPQSLETGQQTRVIKVVPHNRRSAT
ESAARIFKSIQEERKQYDSV

***Cajanus cajan* (L.) Huth**
>CcELF3a3-KK1_009574-Asha
MKRGKDDEKVMGPMFPRLHVNDTERGGPRAPPRNKMALYEQFSIPSQRFN
SGVLPLNPNISSNTVPPASSSQGTVPERNHVFPVHLPPQNPIRRAEKCIS
RQSEGANLSASLEQRKKIDEDDFRVPVYIHSRIGQCNNKSVESFDAKKLT
PTGFRYFGGSVAGQSDCERDPKQFGSSLVNMRKDVRSEIDGLPQVSTRKE
HPLMSVTSISTGENIDTVKHAKVTPNQEFQDCHVSKQNRLRQGDACLRHD
CGVASQSNDIGHNGCLVESARETDKGNAPTVNKISPAEAINDTRHHGTRT
GSSIQRGNLNKSDNASKISMVENLSTAKISPDDVVGIIGQKHFWKARKAI
AT

***Glycine max* (L.) Merr.**
>GmELF3a1-Glyma.04G050200-Wm82.4
MKRGKDDEKVMGPMFPRLHVNDTEKGGPRAPPRNKMALYEQFSIPSQRFNSGVLPLNPNI
SSNTVPPASSSLRTVPERNCVYPVHLPPQRPIHRAEKCNSRQSEGTNLSASLEQRKKVDE
DDFRVPVYFHSRTGQCNDKSVESFNGKKLTPTGSRYFGGSISGQSDCERDPKQFGSSVVN
MRKDVRSEIDVLPQVSTSKEQASMSVRSISTRENIHTLLRQAKVTPNREFQDCHVSKFNR
LQQGETCLQLECGVESRSNDIGDNGCLVESARETDKGNAPTANQTSPADAINDTEHHDTR
MGSPIQRGNLNESDNASKISMVENLSTVRISPDDVVGIIGQKHFWKARRAIANQQRVFAV
QVFELHRLIKQVQQLIAGSPDILLEDGAFLGKSPPKGSTPKKLALEYVVKPRQQNLKRKD
DSEKLNHKMECSAENAVGKTSLSSVKDGSHLSKCTPFPGNQHQTNVAADSGMGPWCFNQS
PPGHPWLIPVMTPSEGLVYKPYPGPGFTGTGCGGGCGPFVPALLGGSFMNPGYGIPTSHQ
GVGVPPDTHPGSHGYLPPYGMPVMNSSMSESVVEQGNQFSALGSHGHNGHLPGGGKANHN
TNNKSSCNLPVQRNGAISHVLKHQTSKDFELQETSASSPSEMAQGLSTGQVAEGRDVLPL
FPMVPAEPESVPQSLETGQHTRVIKVVPHNRRSATASAARIFQSIQEGRKQNDSVX

***Glycine max* (L.) Merr.**
>GmELF3a2-Glyma.17G231600-Wm82.4
MKRGNEEEKLGGPMFPRLHVNDAEKGGPRAPPRNKMALYEQFSIPSQKFNPRLLPRKPNS
SSNIVPPPTQGNGHERSYVYPVRFSSQTPTHRAESYISRQSDDGSRSNTSLVQLERRKKV
DDDIHVYTCSGIDQSNDKTMESVDGKKFTPFGARNFCYSVAVQNDGDKDPTQFSSCSLPV
DLRKDVRNGNEANPHVSSSRRKPKMSVKNNSSGEIIDSLMMQAKVIPNLEDQDYSVPNIS
RLHQDDTCLQKECVAGSQSNDVEHGDDLLNSTRDIDNGNALVPRGCFHSAANQTRPLEAT
NDAEYHDTGTGGPIQKGNFDERDNISKISTVTNLSSLIVSPDDVVGILGQKHFWKARRKI
ANQQSVFAVQVFELHRLIKVQQLIAASPDVLLEDVAFMGKFPLTESPPKNISLEVVVEPQ
QQNPKRKNDSEKLNHKTECSAENAVAKRTSFSSPKNGSHLANHTPFSGTPHQANVASDNK
TSPWCFNQTPGHQWLIPVMSPSEGLVYKPYPGPGFKGTMHGGCGGPFGQAPLSATFMNPA
YQFPASHPVVGVSPFVPPASHTYFAPFGMPVMNQATSGSAVEQVNQFAAQGSHGQNGHSS
VEGADFNTHHNQSSSNLPVQKNGARLHVKKSQALKERGLQGSTRSSPSEMAQGIRAGKIA
DGSDAQSLSLHAVETRQQTQAIKVVPHNRKSATESAARIFQSIQEERKQHDLVX

Glycine max **(L.) Merr.**
>GmELF3a3-Glyma.14G091900-Wm82.4
MKRGNEEEKSAGPMFPRLHVNDAEKGGPRAPPRNKMALYEQFSIPSQRFNPHLPRKPNSS
SNIVPPPTSSTEGNGRERSYISRQSDDGARSNTSLVQLERRKKVDDGIHVYTCSRIDQSN
DKMLESFNGKKLTPFGARNFCYSVAVQNGGDKDTTQFGFLPADMRKDARKGNEANPHVSS
SRQKLKLSVKPKSSGEIIDSLVMQAKVIPNQEDQDYSVPNINRLHQDDACLQKECVAGSQ
SNDIEHGGDLLNSTRDMDNGNALVPRGCFHSAANQTHPLEATNDAEYHDTRTGGPIQKGN
FDESDNISKISTVTNLSSLIVSPDDVVGILGQKHFWKARRKIANQQSVFAVQVFELHRLI
KVQQLIAASPDVLLEDGAFMGKFPLMGSPPKNKNISLEVVVEPQQQNPKQKNDSEKLNHK
TECSAENAVAKKKSFSSPKNGSHHTNHTPFSGTPHQANEASDNKTSPWCFNQAPGHQWLI
PVMSPSEGLVYKPYPGPGFMGTMNGGCGGPFGQAPLGATFMNPAYQFPASHPVVGVSPFV
PPASHAYFPSFGMPVVNQATSGSTVEQVNQFAAQGSHGQNGHSYIEGTDFNTHHNQSSSN
LPVLKNGATLHVKKSQASKERGLQGSTISSPSEMAQGIRAGKIAEGNDAHSLSLQAVETR
QQTQVIKVVPHNRKSATESAARIFQSIQEERKQHDLVX

ELF3b

Lens cuinaris **Medik.**
>LcELF3b-2RBY.7g001120-Redberry
MFCKFGRDKKFFCKFERIECGKKSGMKGEIDEGKDISPMFPRLHVKDAEK
GGPKAPPRNKMALYEQFSIPSQSFAAGGPGSGSLFSLPLRNCTVPSTSSH
LGYNQSIKFCSSKSNAISILSEKSQAYNSRKINLTKLTVSHDLEHMKPAK
QITKVQQDNFINSSKSSLKTLDGKEAFITPSSVQVKSSFYGGVIQNEKDE
DKLARCNLSFSFKSLNSFRKMMNSSGAIELKSSQYGKNRMEEHKDASPID
QKAKEKSPHSLNGFDDTTTESSNSSAKYTNSKSMKDEIKNISVDSLKALQ
GSNGHRHEDHAAFVDKSNFRDQYMEKPATSDVQKCSGELEIGRRSLHGKR
ERSRDEETSRNYDALNKSSSECRFGLDIIPDDVIGLIGEKQFWKTRKTII
NQQRIFFMQVFELHRLIKVQRLIAGSPNILFEDNLVLNKPPTKTPSSEKL
ESDFISEQPLTVFKHDSKSEKASTSEDVKNSAVGNIPFPCVNNISKEHNR
LSYYGNHHLGSLTLASADKNSNSISISKQSSSIVYPPPPNQWLVPVMSPS
EGLVYKPIIGPCPPNAGGIMTPLYGTCGALSLNSGTKDVLDPSLTSFHQK
IGILSGSSLPQLLPPCVPSFMHRSISASAVEQMGQSSGPKNHHSYAELNS
AILYQSPSNMSTQISQVMSRNISTYQSLEDNKELQVSTASSPSKRMKGDE
LPLFPLAPTFWSSPDRDSRVEHQPKVIKALPHNKKSASESAAKIFMSIQD
ERKLL

Pisum sativum **L.**
>PsELF3b1-Psat4g192520-Cameor
MKGAIDEGKEISPMFPRLHVKDAEKGGPKAPPRNKMALYEQFSIPSQSFA
AGGPGSGSLFSLPLRNCTVPTSSSHLGYNQSINFCSSKSNAISILSEKTQ
AYNSRKINLTKLTVSHDLEHMKPAKQITKVQQDNYINSSKSSLKTLDGED
AFITPSSVHLKSSFYGGVVQNEKDEDKLARCNLSFSFKSLNSFRKVMNSS
EAIDLKSSQYGKNRMEEHKDASQIDQKAKEKSPHSLNGFDDTTNESSNSS
AKYTNSKSMKEEIKNISVDSLKALQGSNGHRHEDHAAFVDKNKFRDHCME
KPTMSDVQKCSGELEIGRRSLHGKRERSRDEETSRNYDALNKSSSDCRFG
VDIIPDDVVGLIGEKQFWRTRKTIINQQRNFFMQVFELHRLIKVQRLIAG
SPNLLFEDNLVLNKPPTKTSSPEKLQSDFISEQPLTVFKHDSKSEKASTS
EDVKNSAVGNIPFPCVNNISKEHNRLSYYGNHHLGNLTLVSADKSSSSIC
LSKQSSSIVYPPPPNNG

Pisum sativum **L.**
>PsELF3b2-Psat2g146160-Cameor
MKSFNGENITPTGSRNFGCLVSGKNDSDEDQTQFGSLLVKMRKDVRNKGE
VHLQESISRQKTVMSVKSILTGEIIDSSVRQAENISDEEDQNDACLQQGS
NDIEHGGSLVDSAMDTDNRNNLILRVRFRSTLNQISVPEAADHTEYHDTN
IDSPIENGSSEGSDDLSKNSTLENMPSPRLSPNGVAEISGQQLFGKQDEK
LLINRECLRSKYLNCTN

Cicer arietinum **L.**
>CaELF3b1-Ca11741-Frontier
MKRGKCDDDDDEKVVVGPMFPRLHVNDTTEKGGPRAPPRNKMALYEHFNV
PFQRFNNNSNSSSNSHPLTPSSTLGNDPERSYIVPVNLPSQTATQRAESY

TSHQSNEANMDTLSAQLGQRKKVDGDGSHAYDHSRIGQSNNKTMKSFNGK
KLTPVGARNFCCSVAGENDGDKDQTQFGSLLVKMRKDVNKSRLHQGNACV
QQXXXXQESNDSEHGDGLIDSAMDIDNRNSLVLRSCSHSTVDQTSVLEAT
NDTEYHDTNIDSPMQKGNSEGSDDLSKTSTLESLSSMELSPDGIVQILGQ
KHFWKARRKITTQQRVFAVQVFELHRLIKVQQLIAGSSDLLFEDAAILGK
FPLQASTPKSISLEVIVEPPTQNHKQQDHSEKLNHKLEHSAENGVGKTSF
SSQKYGSHLSNYTPFYGNPHQENVVSRSFNHSPGYQWLIPVMSPSEGLVY
KPYPGPGFTGPMYGGCGPFGPGPPNGAFMNPAYGIPDFHQAVAVSPFIPP
GSYAHFPPHCVPAMNQSASGSVVEQANRFAAQVSHDSNGNSSLVGASFNT
HNQSSCNLTNQKNGATLHITKFQPSIERELQGIPTSRPNEKEIRIEKSAE
GRDALSPSFTAPSVSEEVFQSLETKQKPQVIRVVPHNPRSASVSAARIFQ
SIQEERKQYDLV

***Cicer arietinum* L.**
>CaELF3b2-Ca22545-Frontier
MKGAIDEGKEISPMFPRLHVKDAEKGGPKAPPRNKMALYEQFSIPSSGHG
SGGSLFSLPLRNCTLSTTSTHLGCSTQSIKFCSSNASSILSDKAQAYNSR
MINSTKLMASRDFEHKKPAKQITKDNFISSKNSLKTIDGEEAAFITPGSV
HVKSSCCGLIIQNDKEKLTRENLSCSLKNLNSFRKMMNSNGAIELKSAQC
GKIQMEEHKNSSQIDQKEEEGIPLSLNSFDDMTNASSNSLVKDTNSKSMK
KEHRSMKEETKNISIDSLKTLKGSNGNRHEDHAGFVEKIKLKDHCMEKPA
KSDVERILGELEIGRRSFFGGKRERSKDEETDRNYDALNKPRSAYRFGMD
ISPDDVVGLIGDKQFWNTRKTIINQQRIFLMQVFELHRLIKVQRLIAGSP
HLLFEDKSPIITSSPKKLDDKSEKAATSDDIKNNVVGNIHLPSVNNITKE
HNHLPNYVNHHLGNLALASNDIKNSNSKQSPNIVYPPPPANQWLVPVMSP
SEGLVYKPIIGPCPPNSSLVAPLFGACSSLSLNPGNKDVLDQSLGTNCHQ
KVGIFSSSSLPQLLPTSFMHRSISASAIEQMEQSNYGTKNHQSNAEFMYQ
TPSSNMSTQISQVMSRNISNYQSYEDNKELQISRASSPSKRMKGVDELPL
FPVAPTLWPSSDRDTRVENQSRVIKALPHNAKSASESAARIFKSIQEERK
LL

***Vigna angularis* (Willd.) Ohwi & H.Ohashi**
>VaELF3b-Va11G003150-Jingnong6
MWIVCRSMNRMRGGIEESKEISPMFPRLHVKDAEKGGPKAPPRNKMALYE
QFSIPSQSFAPASASLFPLPLRNYTAHTSSSHIGSNQSIQFNSNAPSILA
EKSQAFNFRRSNLTKYTQDNFINSKNSLKALDGEDVFITAGSAHGKSSCC
SIIQNNKDEDGCNLSCSLKNLNSFRKKVNSPGVVDLKSAQYGKIQMEELT
KVSQNGQKPEEVLSLDGFGDMTDASLISLVQGSSVHTHEEHAAFGDKFNS
RDHHIEKPAASDVHRCSGELEIGRRFFLDKGAGNEDEETSVMHISPDNVV
GAIGEQQFWKARRTIVNQQKIFVMQVFELHRLIKVQRLIAGSPHILLADN
LLLNKPPPKTSTTKKFQSDFAGEKPSSVAKLDSKSVKAPTAEHATNSAVE
KIPIPCVNNITKGHANQLPNYGHHLANLPIASSDTNSKQSPSYVYPPPGN
QWLVPVMSPSEGLVYKPVIGPCPPNAGFMAPIYGTCGSVSFNPLSKDASS
EAALAPSSHQKFGILSGSSLPQFLPPPFIHNPSSMSASAVEQMGQSNGPE
NHNSCGEVNSAILYQSSSNISSPTSQVMSRNISIHQSPKDKEPHRSTASS
PSKRTSAGEVLPLFPLAPTCWQSEDRHTQVELQPRVIKAMPHNPKSASES
AARIFRSIQEERKHL

***Phaseolus vulgaris* L.**
>PvELF3b-010G142900-G19833
MNRMRGGIEESKEISPMFPRLHVKDAEKGGPKAPPRNKMALYEQFSIPSQ
SFAPGSASLFPLPLRNYTGPTSSSHISSNQSIQFTYNAPSIMAEKSQAYN
FRRSNLTKRTQENFINSKNSLKALDGEDVFITSGSAHGKSSSFQNNKDEG
GCNLSCSLKNLNSFRKKVNSPGVVDPKSAQYGKIQMEELTKVSQNGQKPE
EVLSLDGFGDMTDTSLMSLVQGRDSKSMNKEHRSLKDEIRSISVDSLKTL
QGSSVHTHEEHATFGDKFNSRDHHMEKPAAGDVHKCSGELEIGRRFFLDK
GAGNEDEETSAMHISPDNVVGAIGEQQFWKARRTIVNQQKIFVMQVFELH
RLIKVQRLIAGSPHILLIDNLLLNKPPPKTSTTKKFQSDFVGEKPSSVVK
LDSKSVKAPTAEHATNSAVEKIPIPCFNNITKGHTNQPPNFGHHLAKLPI
ASSDTNSKQSPSYVYPPPGNQWLVPVMSPSEGLVYKPVLGPCPPNAGFMA
PIYGTCGSVSFNPLSKDVSSEAALAPSSHQKFGILSGSSLPQFLPPPFIH
NPSSISASAVEQMGQSNGPENHNSCGEVNSAIIYQSSSNISSPTSQVMSR
NISTHHSPKDKEPQRSTASSPSKRMSAAEVLPLFPLAPTFWQTEDRNTQV
ELQPRVIKAMPHNPKSASESAARIFRSIQEERKHL

***Vigna unguiculata* (L.) Walp.**
>VuELF3b-Vigun10g177000-IT97K-499-35
MWIVCRRMNRMRGGIEESKEISPMFPRLHVKDAEKGGPKAPPRNKMALYE
QFSIPSQSFAPASPSLFPLPLRNYTPHTSSSHISSNQSIQFNSSAPSILA
EKSQAYNFRRTNLTKYTQDNFINSKNSLKALDGEDAFITSGSAHGKGSCC
SIIQNNKDEDGCNLSCSLKNLNSFRKKVNSPGVVDLKSAQYGKIQMEELT
KVSQNGQKPEEVLSLDGFGDMTDASLISLVQGRDSKSMNKEHKSLKDEIR
SISVDSLKTLQGNSVHTHEEHAAFGDKFNSRDHHIEKPAASDVHKCSGEL
EIGRRFFLDKGAGNEDEETSVMHISPDNVVGAIGEQQFWKARRTIVNQQK
IFVMQVFELHRLIKVQRLIAGSPHILLADNLLLNKPPPKTSTTKKFQSDF
VSEKPSSVAKLDSKSVKAPTAEHATNSAVEKIPIPCVNNITKGHANQLPN
YGHHLANLPIASSDTNSKQSPSYVYPPPGNQWLVPVMSPSEGLVYKPVIG
PCPPNAGFMAPIYGTCGSVSFNPLSKDASSEAALAPSSHQKFGILSGSSL
PQFLPPPFIHNPSSMSASAVEQMGQSNGPENHNSCGEVNSAIIYQSSSNI
SSPTSQVMSRNISTHHSPKDKEPQRSTASSPSKRGSAGEVLPLFPLAPTF
WQTEDRHTQVELQPRVIKAMPHNPKSASESAARIFRSIQEERKHL

***Vicia faba* L.**
>VfELF3b-Vfaba.4g051760-Tiffany
MKGAIDEGKDISPMFPRLHVKDAEKGGPKAPPRNKMALYEQFSIPSQSIA
AGGPGSGSLFSLPLRNCTVPTTSSHLGYNESIKFCSSKSNAISILSEKTQ
AYNSRKINLTKLTQDNYINSSKSSLKTLDGEDAHITPSSVHVKSSFYGGV
VQNEKDEDKLARCNLSFSFKSLNSFGKVMNSSGAIGMNSPQFGKNQMEEH
KDVSQIDRKAKEKSPHSLNGFDETTNESSNSSAKYTNSKSMKEEIKSVSV
DSLKALQGSNGHRHEDHAAFVDKNNFRDHCMEKPAMSDVQKCSGELEIGR
RSLHGKGERSRGEETSRNYEALNKSSSECRFGLDIIPDDVVGLIGEKQFW
KTRKTIINQQRIFFTQVFELHRLIKVQRLIAGSPNLLFEDNLLLNKPPTK
TSSSEKLQSDFIGEQHLTVFKHDSKFDKASTSEDVKISAVGNIPFPCVNN
ISKEHNRLSYYGNHHLGNLALASADKNSNSISISKQPSSIVYPPPPNQWL
VPVMSPSEGLVYKPIIGPCPPNAGGIMAPLYGACGALSLNPGTKDVLEPS

LTSFHQKIGILSGSSLPQLLPPCVPSFMHRSISASSVEQMGQSSGPKNHH
SYAELNSAILYQSPSNMSTQISQVMSRNISTYQSLEDNKELQASTASSPS
KRMKGDELPLFPLAPTFWSSPDRDSHVEHQSKVIKALPHNKKSASESAAK
IFMSIQDERKLL

***Lupinus angustifolius* L.**
>LaELF3b1-R12006-Tanjil
MKRGKAEKVGPMFPRLHVNDTEKGGPRAPPRNKMALYEHFTIPSRGSSNK
GTGSERSYIIPGNLPSQMPTQRAESYISHQFNGASQNTSSAQLEQRNKVD
EDDFMVPVYVDSRIGQYNDKRLVNFEMEKLTSMSSRNFGCSIEVQKDGGR
HRKQLDSPPINIREDASSESEGLPQVSESRELHVKNISTIETIKSLARQA
KVIQNQEYQDSPVEATHDAEFHDTRTGSLVQKGSSDDGDDVSKISSTENL
SSLKASPDDVVGVLGQKRFWKARRKFVNQQSVFAVQVFELHRLLKVQKLI
AGSPDLLLEDGSFMRNSLPKRSTPNLAVNVVVKPQIQNLKRKDDSEKLNH
NKMEYSAENVVGKRSCSIPENGCHLSNYTSFSGTPHQTKVAADNRMGSWG
FNHSPGHQWLIPVMTPSEGLVYKPYPGPAFQETMCGRGCEPFGPGPPGGT
FMNPSYGVPPHIPPGSLAYFHPYGMPVMNQSVSGSADEQVNHLAAQGSRG
QNGHSSIEEANFSTHNQSSYNLPAQKNGAISHVKKFQASKKSEIQGSTAS
SLIEKAHGIRQRQIAEGRNAHSLSHMATLIPEGVLQPLETGKQTRVIKVV
PHNRRSATESAARIFQSIQEERKQCDLL

***Lupinus angustifolius* L.**
>LaELF3b2-R13664-Tanjil
MKGIIDEGKEISPMFPRLHVKDAEKGGPKPPPRNKMALYEQFNIPSQSIA
SGSTSLYPLPLRKCTIPSTSSHVSSNQTIQFCTSSAASVVAENIQVYDSR
KINLNKLMQHNFINSKKSLKIFDGEDISIASSSDFGKNSSCSMIQNDKLD
NCNLIYPLKSPSSLRKKVSSPETIALEFAQYGKNLMEEHFKMSRKGWKPE
EESANPIDGFCGMADSSFLSLNKDKNFKSSMKEHRFLKEETTSISKDCLK
TLQGNNAEAREEHDEAFANIVDLQDNCMKKTADNDVYKCPDELEIGRRCL
LSKRDRNKDEETCRDYDALNRPTSECKVGTDISPDSVLGVIGLKKFWKAR
KTIINQQRMFFMQVFELHRLIKVQKTIAASPHLLLEDNLVSNKQSLKPSA
IKKIQSDYQQTTSIVKLNTKSEKPPTAEHAKNIAFRKIPPLPCLNNISKG
LPYFVHDLRNPALCLPDINIKQSPSCVFPPPGNQWLVPVMSPSEGLVYKP
MAGPCPPNPGFMPPPVHNAFSTMAFNLGSKDATDAAALSSGSHQRTGIPS
GSSLPNSLPPPFMIPPMLPVSAVEHIWQSNGPEEANSAILYQSSSNLSSQ
ASRALSRNVPTSHHSLKEKESQTSTATSPPKRVKGDVLPLFPVAPTLWPS
TKDKNKNVENQPRIIKAMPHNPKSATESAARIFRSIQEERKFL

***Cajanus cajan* (L.) Huth**
>CcELF3b-KK1_000279-Asha
MQVFELHRLIKVQRLIAGSPHILLEDNLLLNKPPPKTSTTKKLQSDDFVG
EKPSSVVKLDSKSGNAPPTEHVTNNAFGKIPIPCVNNITKGHANQLPNYS
HHLGNLAVASADSNSKQSPSYVYPPPGNQWLVPVMSPSEGLVYKPIIGPC
PPNAGFMAPIYGTCGTVNFNPGSKDASLDAALAPSSQQKFGLLSGPSLPQ
FLPPPFVPPSSISASAVEQMGQSNGPENHHSCGEVNSAILHQSSSNISSP
TSQVMSINISTYHSVKDKELQRSTASSPSKRTSGELLPLFPLAPTFCHTE
DRSTQVEQQPRVIKAMPHNPKSATESAARIFRSIQEERKHL

***Glycine max* (L.) Merr.**
>GmELF3b1-Glyma.07G013500-Wm82.4
MMNRMSGGIEEDKEISPMFPRLHVKDAERGGPKAPPRNKMALYEQFSLPSQNFAPGSASL
FPLPLRNYTGPTSSSHISSNQSIQFCTSSMPSILAEKSQTYNSRKTNLTKFMQDDFINSK
NSLRTLDGEDTFITCGSAHRKNSSCSIIQNFKDEDKLSHSNLSGSLKSPNSFRKVNSPGA
VDLKSAQYGKNRMEDHTEVSQNGQKPEEVLPHSLDGFGDMTDASLISLVKGKNSKSMNKE
HRSLKEEKRSILIDSLKTSQGSSVRTHEELAVFEDQVNSRDHHIEKPTANDVHKCAGELE
IGRRCFLDKVDMNEDEETYRHSDAVSKVNSECTLAMDISPDNVVGAIGEQQFWKVRRTII
NQQKIFVKQVFELHRLIKVQRLIAGSPHVLLEDNLLLNKPPPKTSTSKKFQSDFASQKPS
SVVKLDNKSVKVNTAEKATNSAVGKIPIPCISNTTKGHANQLPNYGHHLGNPALASADSN
SKQSPSYVYPPPGNQWLVPVMSPSEGLVYKPIIWPCPPNAGFMAPIYGSCGTVSFNPLSK
DASSDAALAPSSHQKLGILSGSSLPQFLPPSFMNHPSSISASAVEQMGQSNGPENHHSWG
EVNSAILFQSSSNMSSPTSQVMPRNISTYHHSLQDKELHRSTASSPSKRVSGEALPLFPL
APTCWQSEDTNTQVEQQPRVIKAMPHNPKTASETAAKIFRSIQEERKHLX

***Glycine max* (L.) Merr.**
>GmELF3b2-Glyma.08G197500-Wm82.4
MMNRTRGGIEEGKEISPMFPRLHVKDAEKRGPKAPPRNKMALYEQFSIPSQNFAPGSTSL
FPLPLRNYTGPTSSSHISSNQSIQFCTSSMPSILAEKSQAYNSRKTNLTKFTQDDFINSK
NSLKALDGEDAFISSGSAHRKNSYCSIIQNNKDEGKLSCSNLSCSHKRLNSIRKVNSPGA
VDLMSEQYEKNQIEEHTEVSQIGQKPEEVLPHSLDGFGDMTDASLISLEKGKNSKSMNKE
HRSLKEENRSISVDSLKTLQGSSVRTQEELAAFRDQIKSRDHHIEKPAASELHKCSGELE
IGRRCFLDKVNRNEDEETYSHYDAVNKYNSECTSVMDISPDNVVGAIGEQQFWKVRRTII
NQQKIFVKQVSELHRLIKVQRLIAGSPHILLEDNLLHNKPPQKTSTTQKFQSDFAGKKPS
SVVKLDNKSVKVNTDEKATNSAVGKIPIPCISNITKGHANQLPNYGHHLGNLALGSADSN
SKQPPSYVYPPPGNQWLVPVMSPSEGLVYKPIIGPCPPNAGFMAPIYGSCGTMSFNPLSK
DASSDATLAPSSHQKLGIISGSSLSQLPSFMNHPSSLSASAIEQMGQSNGPENHHSCGEV
NSAILHQSSSNMSSPTSQMMSRNISTYHHSLKDKELQRSTASSPSKRMSGEALPLFPLAP
TFWQSEDRNTQVEHRPRVIKAMPHNPKTASESAARIFRSIQEERKHLX

Supplementary material S3. Sequences of primers and entire *LcELF3a* (Lcu.2RBY.3g063730) gene in lentil used in this study

Primers for sequencing:

LcEFL3-F1: CAGGCAGTCCAAAACAGTTAGG 22 bp, 50%GC, Tm=54.8C
LcEFL3-R1: CTGTTCAAGTGTCCCAACCATG 22 bp, 50%GC, Tm=54.8C
(RevCom): CATGGTTGGGACACTTGAACAG
Amplicon size = 863 bp

LcEFL3-F2: CACTGGGGCATGTTTCCTTGTA 22 bp, 50%GC, Tm=54.8C
LcEFL3-R2: GGAGTAGATCCATCTGGAAGAG 22 bp, 50%GC, Tm=54.8C
(RevCom): CTCTTCCAGATGGATCTACTCC
Amplicon size = 853 bp

>JX946295.1 Lens culinaris. Early flowering 3a gene (ELF3a), complete cds

ATG, 'Start' codon; TAG, 'Stop' codon.
Yellow – Exons; and no colours – Introns or UTRs

GCGGCCCAGTAAAGATGTGAGTAGGGAGGTTAGGATGAAAAGAGGGAGTGATGATGAGAAAATG
ATGGGGCCATTATTTCCTAGATTACATGTTGGTGATACAGAGAAAGGAGGGCCTAGAGCACCAC
CTAGGAATAAAATGGCTTTATATGAGCAGTTTAGTATTCCTTCTCAAAGGTTTAACTTGCCACT
ACACCCTAATAATTCGACCAATTCGGTTCCCCCGGCTTCCTCGAGCCAGGTAATTTTTAGAATC
TCGGGCTTTTTTTTTATATCTGGAGATTAACAATTTTGATGGAAACAATGGTTCTGGATAGAAC
AGTTGATTCATGTATCTGATCCCACTTAGTGGGATAAGACTTGGTTGTGCTGTTGTTGTTGTTG
TGGATTTTCTTATACTAATTATTATCGTTATTATTAGTATTATGTTTAGTTCATTGTAGCATTA
GAATAGTTTAATTCTAGTTTTAAGATTGTGTGAGAAATAGGTTTTTTTTGTTATTTTCTGAATG
TTTGTTTTTTACATGTTGCAGGGGACTGTCCATGAGCGAAACTATATTTTTCCTGGTCATTTGA
CGCCTGAAACACTTATTCGTCAGGCTGGAAAACATCTCTCTCGCCAATCAAAAGGGGCAAATTT
GAATGGTTCTATAGCACAAATTGAACATAGAAAGAAGGTTGA***T***GAAGATGACTTTAGGGTTCC***T***
GTATACGTTCGTTCAAATATTGGTCAATCTAATGAGAAAAGGCCTGAGAGTTTTGATGGAAAA
GACTCCCTTCTACAGGCTCTAGGTATTTTGGTTTTTTGAAGCCAGGTAAGATTGATCGGGAGAG
GGAGCTGATACAGAATGGCTCCACAGTTGTTAATGCAGGGACAGACGTGAGAAATGAGATTGAT
GGTCCTCCGCAAGTGAGTCCAAATAAGGAGCATCCGGTTACGTCTGCCAGGAACGCATCGACTG
GAGAACGTGTTGATGCCTTAGTAAGACAAGTCAAAGTGACTCCAAATCAAGAGGTTCAAGATCG
TCGCGTGTTCAAACATAGCAGTTTACGTCAAGGTGATGCACGCTTACGACAAGATTGCCGAGCT
GAGTCTCAATCCAATGGACATGGACAAAGTGACGGTCTCCTTGAATCTACAAGGGAGGTAGACA
CGAGTAATGGCCCTATAGTAAACCAAATCAGTCCAACCCAGGCTATCAATGACACTGAATATCA
TGATACCGGGACAGGCAGTCCAAAACAGTTAGGAAATTTAAACAAAAATGACAACATTTCCAAG
ATCTCCAGGGTAGAAAATTTGTCAACTGTGAAAATTTCTCCTGATGATGTTGTTGCAGTAATAG
GCCAAAAACATTTCTGGAAAGCCAGAAAAGCAATTGCCAAGTAAGTTTCCTTGCT**T**GTCAGGTT
CTTTTATGATTTTAAATAGGAATGTTTGGTAGTCCGATGATTTGATTTTTGTTATTTTGGTGGT
TTATTGGTATTTTCTCCTACCTTCTTTTTTATATTTTCTTGCATTTATCATTAGCCAGGGCTGG
GTTTGAAAATATATATGAAAAAATCCTTTGAAGAGAAGAAATGTAGGACCAGGTAATGTGAATT

ATAGGGACACTCCTATCTAACGTTGCCATTTTTTCCCTTACCTTTTAAAGCATCTTTTATGCAT
CCTTAATTAAAAACCAGGGATGTAAGTTGGATAGTTACATTTCAATTTTCAATCAAGGAGTTGT
ATGTATGGCTTTGAAGAAAGGAGATTTTAAGTCCTGGGATCCTCGCTGAATTGGTTTTATTAGT
TTTATTTCTGCATTCAAAGTCCTTACTAATGTTTTAATGGTGGGTACTGGTTACTACAGGGCGG
TGAGCTTTAATATTATAATATGTTGGAGGGGTCCTTCTGATAGCAGCCGTCGTATACCTTACTG
TCTTCTAGCTACTGGATTTTGGATGTATTCCTGTCTTGGTTTCCTCTTGCTTAATTCTCCATGT
CACCACTGGGGCATGTTTCCTTGTACTACACTCAAGGACTTGAGCTAAGAAGGGGACCAAGTAA
TTCAGGATGAACCCGGGATGCATGGTTGGGACACTTGAACAGTGTATGAGCCCTCAATAGATGT
TTTGATAGTTAGCGGATTTCCCCAACTTATCTAATGTCTTGGTTTTCTGTGTTTTGGAACATTT
TGGGTAAGGGATTTTTTTAATAAACAGACACATTAGCTACTTGAGGTGTCCTGCTATCTATAGC
TCCAACAGATTTCATATGATTAATATTGTGTGATTGAAGATTCTTTGATTTTTGTAATGTGTGG
TGTTTAAACTGGTCCTTTCAACCTTATCATATCTAACTTAAAATGCTTTATTTTCTATTTTATT
TTGATTGTATGCGTCATAAGTCCTCTTTTGATCCCCATGTTATTAATGTTGATTGAAAAATAGC
CTGTTGAACCTGTTACCCGCTGTGATTGATGATCCTTCAATTTTCGTTTATAAATTGGTGTTTC
TAACATTATTTTTGTAGTTCCAAATGCTCTATCTTTAATTTCATTTCAATTGTCTGTGTTTTAA
GAATTTTCTTGATCCAAATTCTATTCCTCTCTACAGTCAACAGAGAGTGTTTGCAGTACAAGTG
TTTGAGTTGCATAGACTGATAAAGGTAAATTATCTTTGACATTGATCAGTGCTTTGTACTAGTC
CAAAATGAGGAATGCTCTCGCAACCTTGAGCCTGCCTGTATTGATTACTTATTTTCATTTAAGC
AGGTCCAACAACTGATTGCCGGATCGCCAGATCTATTGTTTGACGATGGTGCTTTTTTAGGAAA
GTCTCTTCCAGATGGATCTACTCCTAAAAAACTCCCATTGGAATATGTTGTAAAAACTCGGCTA
CAAAATCTTAAGCGCAAAGTTGATTCTGAAAAGATAAATCAAAACATGGAATGTTCTGCAGAGA
ATGCTGTTGGTAAAACATCTATTTCGTCTGTGAAAAATACGAGCCACCTTTCTAGTTCCATGCC
TTTTGCCGGAAATCCACACCAAGGAAATATGGCTGCTGATAATGGGATGGGTCCCTGGTGTTTC
AATCAGTCACCTGGGCATCAGTGGTTAATTCCCGTGATGTCTCCTTCTGAAGGGCTCGTCTACA
AGCCATATCCTGGGCCTGGATTTACCGGAACAAATTTTGGAGGATGTGGGCCCTATGGGGCTTC
TCCTTCGGGTGGCACTTTTATGAATCCTTCCTATGGAATCCCACCTCCACCAGAGATTCCTCCA
GGCAGCCATGCTTACTTCCCTCCGTATGGCGGCATGCCAGTTATGAAAGCTGCAGCTTCAGAGT
CAGCTGTTGAACATGTGAACCAATTCTCCGCACATGGGCAAAATCACCGTTTATCTGAAGACGA
AGATAATTGTAACAAACATAATCAAAGCTCATGCAATTTACCAGCTCAGAGAAATGAAGATACA
TCACATGTCATGTATCATCAGAGATCGAAGGAGTTTGATTTGCAGATGAGTACCGCAAGTAGTC
CTAGTGAAATGGCACAAGAAATGAGCACGGGGCAAGTTGCTGAAGGAAGAGATGTACTACCTCT
TTTCCCTATGGTTTCAGCAGAACCAGAGAGTGTACCTCATTCTCTCGAAACAGGACAGCAAACT
CGAGTTATCAAAGTGGTACCTCATAACCGAAGATCTGCAACTGAATCAGCAGCTAGAATTTTCC
AATCAATTCAAGAAGAGAGAAAACAATACGATGCACCCTAGTGATATTTATTGGCATGGAGGAT
CCATTCAAGTGCAGTCGCCTAGTCTGAAACC

Supplementary material S4. Details of allele-specific primers used for ASQ genotyping

LcEFL3a-SNP3
TTCTCCTACCTTCTTTTTTATATTTTCTTGCATTTATCATTAGCCAGGGCTGGGTTTGAAAATA
TATATGAAAAWATCCTTTGAAGAGAAGAAATGTAGGACCAGGTAATGTGAATTATAGGGACACT
CCTATCTAACGTTGCCATTTTTTCCCTTACCTTTTAAAGCATCTTTTATGCATCCTTAATTAAA

SNP3: [**W**]=[**A**]or[**T**]
[**A**], Haplotype A
[**T**], Haplotype B

Allele-specific primer design
LcEFL3a-SNP3-F1: CATTTCTTCTCTTCAAAGGATTT 23 bp, 30%GC, Tm=56C
(RevCom): AAATCCTTTGAAGAGAAGAAATG

LcEFL3a-SNP3-F2: CATTTCTTCTCTTCAAAGGATAT 23 bp, 30%GC, Tm=56C
(RevCom): ATATCCTTTGAAGAGAAGAAATG

LcEFL3a-SNP3-R: CAGGGCTGGGTTTGAAAAT 19 bp, 47%GC, Tm=55C
Amplicon size = **52 bp.**

Allele-specific primer order
LcEFL3a-SNP3-F1: GTCCTTGCGAAGGCATCCCATTTCTTCTCTTCAAAGGAT**T**T
LcEFL3a-SNP3-F2: GTCCTTGCGAAGGCCAACCATTTCTTCTCTTCAAAGGAT**A**T
LcEFL3a-SNP3-R: CAGGGCTGGGTTTGAAAAT

Supplementary material S5. Details of primer sequences with amplicon sizes used for qPCR

Gene identifications are present in Material and Method section of the paper

Name	Sequence, 5'-3'	Amplicon
LcELF3a-Fq	GTAGACACGAGTAATGGCCCTA	104
LcELF3a-Rq	CCTAACTGTTTTGGACTGCCTG	
LcELF3b-Fq	GGATATGAACCGTTTGGACACAG	106
LcELF3b-Rq	GAGGAATGAATGGTGACATCGC	
LcFTa1-Fq	TCGTCAGCAGTGTAGACAAAGG	129
LcFTa1-Rq	CTCTCCCTCTGACAGTTGAAGA	
LcFTa2-Fq	TGATCTACTACACCCTGGTGTTA	129
LcFTa2-Rq	TAATGAACCCAAAACTAGTCCCG	
LcFTb1-b2-Fq	GACACACTATATTACCACCTGGT	130
LcFTb1-b2-Rq	CTTCCACCACAACCATTTTGTC	
LcFTc-Fq	CTGGGCAGAGACACAGTTTTTG	134
LcFTc-Rq	CTTCCACCGCAACCACGC	
LcElf4A-III-Fq	TCATCGGATTGGTCGTTCTGGA	126
LcElf4A-III-Rq	GGCATCTCATCAATCTGGGTAC	

Supplementary material S6. Sequence alignment of fragments in *LcELF3a* gene with identified SNPs and InDel in two haplotypes, A and B.

```
JX946295          CGGGTAGAAAATTTGTCAACTGTGAAAATTTCTCCTGATGATGTTGTTGCAGTAATAGGC
Niva-95           CGGGTAGAAAATTTGTCAACTGTGAAAATTTCTCCTGATGATGTTGTTGCAGTAATAGGC
Flip92-36L        CGGGTAGAAAATTTGTCAACTGTGAAAATTTCTCCTGATGATGTTGTTGCAGTAATAGGC
ILL-1552          CGGGTAGAAAATTTGTCAACTGTGAAAATTTCTCCTGATGATGTTGTTGCAGTAATAGGC
Vekhovskaya       CGGGTAGAAAATTTGTCAACTGTGAAAATTTCTCCTGATGATGTTGTTGCAGTAATAGGC
Krapinka          CGGGTAGAAAATTTGTCAACTGTGAAAATTTCTCCTGATGATGTTGTTGCAGTAATAGGC
Flip96-48L        CGGGTAGAAAATTTGTCAACTGTGAAAATTTCTCCTGATGATGTTGTTGCAGTAATAGGC
Syrian-Local      CGGGTAGAAAATTTGTCAACTGTGAAAATTTCTCCTGATGATGTTGTTGCAGTAATAGGC
                  ************************************************************

JX946295          CAAAAACATTTCTGGAAAGCCAGAAAAGCAATTGCCAAGTAAGTTTCCTTGCTTGTCAGG
Niva-95           CAAAAACATTTCTGGAAAGCCAGAAAAGCAATTGCCAAGTAAGTTTCCTTGCTTGTCAGG
Flip92-36L        CAAAAACATTTCTGGAAAGCCAGAAAAGCAATTGCCAAGTAAGTTTCCTTGCTTGTCAGG
ILL-1552          CAAAAACATTTCTGGAAAGCCAGAAAAGCAATTGCCAAGTAAGTTTCCTTGCTTGTCAGG
Vekhovskaya       CAAAAACATTTCTGGAAAGCCAGAAAAGCAATTGCCAAGTAAGTTTCCTTGCTTGTCAGG
Krapinka          CAAAAACATTTCTGGAAAGCCAGAAAAGCAATTGCCAAGTAAGTTTCCTTGCTTGTCAGG
Flip96-48L        CAAAAACATTTCTGGAAAGCCAGAAAAGCAATTGCCAAGTAAGTTTCCTTGCTTGTCAGG
Syrian-Local      CAAAAACATTTCTGGAAAGCCAGAAAAGCAATTGCCAAGTAAGTTTCCTTGCTTGTCAGG
                  ************************************************************

JX946295          TTCTTTTATGATTTTAAATAGGAATGTTTGGTAGTCCGATGATTTGATTTTTGTTATTTT
Niva-95           TTCTTTTATGATTTTAAATAGGAATGTTTGGTAGTCCGATGATTTGATTTTTGTTATTTT
Flip92-36L        TTCTTTTATGATTTTAAATAGGAATGTTTGGTAGTCCGATGATTTGATTTTTGTTATTTT
ILL-1552          TTCTTTTATGATTTTAAATAGGAATGTTTGGTAGTCCGATGATTTGATTTTTGTTATTTT
Vekhovskaya       TTCTTTTATGATTTTAAATAGGAATGTTTGGTAGTCCGATGATTTGATTTTTGTTATTTT
Krapinka          TTCTTTTATGATTTTAAATAGGAATGTTTGGTAGTCCGATGATTTGATTTTTGTTATTTT
Flip96-48L        TTCTTTTATGATTTTAAATAGGAATGTTTGGTAGTCCGATGATTTGATTTTTGTTATTTT
Syrian-Local      TTCTTTTATGATTTTAAATAGGAATGTTTGGTAGTCCGATGATTTGATTTTTGTTATTTT
                  ************************************************************

                 SNP1    SNP2
JX946295          GGTGGTTTATTGGTATTTTCTCCTACCTTCTTTTTTATATTTTCTTGCATTTATCATTAG
Niva-95           GGTGGTTTATTGGTATTTTCTCCTACCTTCTTTTTTATATTTTCTTGCATTTATCATTAG
Flip92-36L        GGTGGTTTATTGGTATTTTCTCCTACCTTCTTTTTTATATTTTCTTGCATTTATCATTAG
ILL-1552          GGCGGTTAATTGGTATTTTCTCCTACCTTCTTTTTTATATTTTCTTGCATTTATCATTAG
Vekhovskaya       GGCGGTTAATTGGTATTTTCTCCTACCTTCTTTTTTATATTTTCTTGCATTTATCATTAG
Krapinka          GGCGGTTAATTGGTATTTTCTCCTACCTTCTTTTTTATATTTTCTTGCATTTATCATTAG
Flip96-48L        GGTGGTTTATTGGTATTTTCTCCTACCTTCTTTTTTATATTTTCTTGCATTTATCATTAG
Syrian-Local      GGTGGTTTATTGGTATTTTCTCCTACCTTCTTTTTTATATTTTCTTGCATTTATCATTAG
                  ** **** ****************************************************

                                                SNP3
JX946295          CCAGGGCTGGGTTTGAAAATATATATGAAAAATTCCTTTGAAGAGAAGAAATGTAGGACC
Niva-95           CCAGGGCTGGGTTTGAAAATATATATGAAAAATTCCTTTGAAGAGAAGAAATGTAGGACC
Flip92-36L        CCAGGGCTGGGTTTGAAAATATATATGAAAAATTCCTTTGAAGAGAAGAAATGTAGGACC
ILL-1552          CCAGGGCTGGGTTTGAAAATATATATGAAATATTCCTTTGAAGAGAAGAAATGTAGGACC
Vekhovskaya       CCAGGGCTGGGTTTGAAAATATATATGAAATATTCCTTTGAAGAGAAGAAATGTAGGACC
Krapinka          CCAGGGCTGGGTTTGAAAATATATATGAAATATTCCTTTGAAGAGAAGAAATGTAGGACC
Flip96-48L        CCAGGGCTGGGTTTGAAAATATATATGAAAAATTCCTTTGAAGAGAAGAAATGTAGGACC
Syrian-Local      CCAGGGCTGGGTTTGAAAATATATATGAAAAATTCCTTTGAAGAGAAGAAATGTAGGACC
                  ****************************** *****************************
```

```
JX946295          AGGTAATGTGAATTATAGGGACACTCCTATCTAACGTTGCCATTTTTTCCCTTACCTTTT
Niva-95           AGGTAATGTGAATTATAGGGACACTCCTATCTAACGTTGCCATTTTTTCCCTTACCTTTT
Flip92-36L        AGGTAATGTGAATTATAGGGACACTCCTATCTAACGTTGCCATTTTTTCCCTTACCTTTT
ILL-1552          AGGTAATGTGAATTATAGGGACACTCCTATCTAACGTTGCCATTTTTTCCCTTACCTTTT
Vekhovskaya       AGGTAATGTGAATTATAGGGACACTCCTATCTAACGTTGCCATTTTTTCCCTTACCTTTT
Krapinka          AGGTAATGTGAATTATAGGGACACTCCTATCTAACGTTGCCATTTTTTCCCTTACCTTTT
Flip96-48L        AGGTAATGTGAATTATAGGGACACTCCTATCTAACGTTGCCATTTTTTCCCTTACCTTTT
Syrian-Local      AGGTAATGTGAATTATAGGGACACTCCTATCTAACGTTGCCATTTTTTCCCTTACCTTTT
                  ************************************************************

JX946295          AAAGCATCTTTTATGCATCCTTAATTAAAAACCAGGGATGTAAGTTGGATAGTTACATTT
Niva-95           AAAGCATCTTTTATGCATCCTTAATTAAAAACCAGGGATGTAAGTTGGATAGTTACATTT
Flip92-36L        AAAGCATCTTTTATGCATCCTTAATTAAAAACCAGGGATGTAAGTTGGATAGTTACATTT
ILL-1552          AAAGCATCTTTTATGCATCCTTAATTAAAAACCAGGGATGTAAGTTGGATAGTTACATTT
Vekhovskaya       AAAGCATCTTTTATGCATCCTTAATTAAAAACCAGGGATGTAAGTTGGATAGTTACATTT
Krapinka          AAAGCATCTTTTATGCATCCTTAATTAAAAACCAGGGATGTAAGTTGGATAGTTACATTT
Flip96-48L        AAAGCATCTTTTATGCATCCTTAATTAAAAACCAGGGATGTAAGTTGGATAGTTACATTT
Syrian-Local      AAAGCATCTTTTATGCATCCTTAATTAAAAACCAGGGATGTAAGTTGGATAGTTACATTT
                  ************************************************************

                                                              SNP4
JX946295          CAATTTTCAATCAAGGAGTTGTATGTATGGCTTTGAAGAAAGGAGATTTTAAGTCCTGGG
Niva-95           CAATTTTCAATCAAGGAGTTGTATGTATGGCTTTGAAGAAAGGAGATTTTAAGTCCTGGG
Flip92-36L        CAATTTTCAATCAAGGAGTTGTATGTATGGCTTTGAAGAAAGGAGATTTTAAGTCCTGGG
ILL-1552          CAATTTTCAATCAAGGAGTTGTATGTATGGCTTTGAAGAAAGGAAATTTTAAGTCCTGGG
Vekhovskaya       CAATTTTCAATCAAGGAGTTGTATGTATGGCTTTGAAGAAAGGAAATTTTAAGTCCTGGG
Krapinka          CAATTTTCAATCAAGGAGTTGTATGTATGGCTTTGAAGAAAGGAAATTTTAAGTCCTGGG
Flip96-48L        CAATTTTCAATCAAGGAGTTGTATGTATGGCTTTGAAGAAAGGAGATTTTAAGTCCTGGG
Syrian-Local      CAATTTTCAATCAAGGAGTTGTATGTATGGCTTTGAAGAAAGGAGATTTTAAGTCCTGGG
                  ******************************************** ***************

                                         SNP5
JX946295          ATCCTCGCTGAATTGGTTTTATTAGTTTTATTTCTGCATTCAAAGTCCTTACTAATGTTT
Niva-95           ATCCTCGCTGAATTGGTTTTATTAGTTTTATTTCTGCATTCAAAGTCCTTACTAATGTTT
Flip92-36L        ATCCTCGCTGAATTGGTTTTATTAGTTTTATTTCTGCATTCAAAGTCCTTACTAATGTTT
ILL-1552          ATCCTCGCTGAATTGGTTTTATTATTTTTATTTCTGCATTCAAAGTCCTTACTAATGTTT
Vekhovskaya       ATCCTCGCTGAATTGGTTTTATTATTTTTATTTCTGCATTCAAAGTCCTTACTAATGTTT
Krapinka          ATCCTCGCTGAATTGGTTTTATTATTTTTATTTCTGCATTCAAAGTCCTTACTAATGTTT
Flip96-48L        ATCCTCGCTGAATTGGTTTTATTAGTTTTATTTCTGCATTCAAAGTCCTTACTAATGTTT
Syrian-Local      ATCCTCGCTGAATTGGTTTTATTAGTTTTATTTCTGCATTCAAAGTCCTTACTAATGTTT
                  ************************ ***********************************

JX946295          TAATGGTGGGTACTGGTTACTACAGGGCGGTGAGCTTTAATATTATAATATGTTGGAGGG
Niva-95           TAATGGTGGGTACTGGTTACTACAGGGCGGTGAGCTTTAATATTATAATATGTTGGAGGG
Flip92-36L        TAATGGTGGGTACTGGTTACTACAGGGCGGTGAGCTTTAATATTATAATATGTTGGAGGG
ILL-1552          TAATGGTGGGTACTGGTTACTACAGGGCGGTGAGCTTTAATATTATAATATGTTGGAGGG
Vekhovskaya       TAATGGTGGGTACTGGTTACTACAGGGCGGTGAGCTTTAATATTATAATATGTTGGAGGG
Krapinka          TAATGGTGGGTACTGGTTACTACAGGGCGGTGAGCTTTAATATTATAATATGTTGGAGGG
Flip96-48L        TAATGGTGGGTACTGGTTACTACAGGGCGGTGAGCTTTAATATTATAATATGTTGGAGGG
Syrian-Local      TAATGGTGGGTACTGGTTACTACAGGGCGGTGAGCTTTAATATTATAATATGTTGGAGGG
                  ************************************************************

                                InDel
JX946295          GTCCTTCTGATAGCAG-CCGTCGTATACCTTACTGTCTTCTAGCTACTGGATTTTGGATG
Niva-95           GTCCTTCTGATAGCAG-CCGTCGTATACCTTACTGTCTTCTAGCTACTGGATTTTGGATG
Flip92-36L        GTCCTTCTGATAGCAG-CCGTCGTATACCTTACTGTCTTCTAGCTACTGGATTTTGGATG
ILL-1552          GTCCTTCTGATAGCAGACCGTCGTATACCTTACTGTCTTCTAGCTACTGGATTTTGGATG
Vekhovskaya       GTCCTTCTGATAGCAGACCGTCGTATACCTTACTGTCTTCTAGCTACTGGATTTTGGATG
Krapinka          GTCCTTCTGATAGCAGACCGTCGTATACCTTACTGTCTTCTAGCTACTGGATTTTGGATG
Flip96-48L        GTCCTTCTGATAGCAG-CCGTCGTATACCTTACTGTCTTCTAGCTACTGGATTTTGGATG
Syrian-Local      GTCCTTCTGATAGCAG-CCGTCGTATACCTTACTGTCTTCTAGCTACTGGATTTTGGATG
                  **************** *******************************************
```

```
JX946295          TATTCCTGTCTTGGTTTCCTCTTGCTTAATTCTCCATGTCACCACTGGGGCATGTTTCCT
Niva-95           TATTCCTGTCTTGGTTTCCTCTTGCTTAATTCTCCATGTCACCACTGGGGCATGTTTCCT
Flip92-36L        TATTCCTGTCTTGGTTTCCTCTTGCTTAATTCTCCATGTCACCACTGGGGCATGTTTCCT
ILL-1552          TATTCCTGTCTTGGTTTCCTCTTGCTTAATTCTCCATGTCACCACTGGGGCATGTTTCCT
Vekhovskaya       TATTCCTGTCTTGGTTTCCTCTTGCTTAATTCTCCATGTCACCACTGGGGCATGTTTCCT
Krapinka          TATTCCTGTCTTGGTTTCCTCTTGCTTAATTCTCCATGTCACCACTGGGGCATGTTTCCT
Flip96-48L        TATTCCTGTCTTGGTTTCCTCTTGCTTAATTCTCCATGTCACCACTGGGGCATGTTTCCT
Syrian-Local      TATTCCTGTCTTGGTTTCCTCTTGCTTAATTCTCCATGTCACCACTGGGGCATGTTTCCT
                  ************************************************************

                    SNP6
JX946295          TGTACTACACTCAAGGACTTGAGCTAAGAAGGGGACCAAGTAATTCAGGATGAACCCGGG
Niva-95           TGTACTACACTCAAGGACTTGAGCTAAGAAGGGGACCAAGTAATTCAGGATGAACCCGGG
Flip92-36L        TGTACTACACTCAAGGACTTGAGCTAAGAAGGGGACCAAGTAATTCAGGATGAACCCGGG
ILL-1552          TGCACTACACTCAAGGACTTGAGCTAAGAAGGGGACCAAGTAATTCAGGATGAACCCGGG
Vekhovskaya       TGCACTACACTCAAGGACTTGAGCTAAGAAGGGGACCAAGTAATTCAGGATGAACCCGGG
Krapinka          TGCACTACACTCAAGGACTTGAGCTAAGAAGGGGACCAAGTAATTCAGGATGAACCCGGG
Flip96-48L        TGTACTACACTCAAGGACTTGAGCTAAGAAGGGGACCAAGTAATTCAGGATGAACCCGGG
Syrian-Local      TGTACTACACTCAAGGACTTGAGCTAAGAAGGGGACCAAGTAATTCAGGATGAACCCGGG
                  ************************************************************
```

Supplementary material S7. Histogram of 66 studied lentil accessions for days to flowering for three studied years.

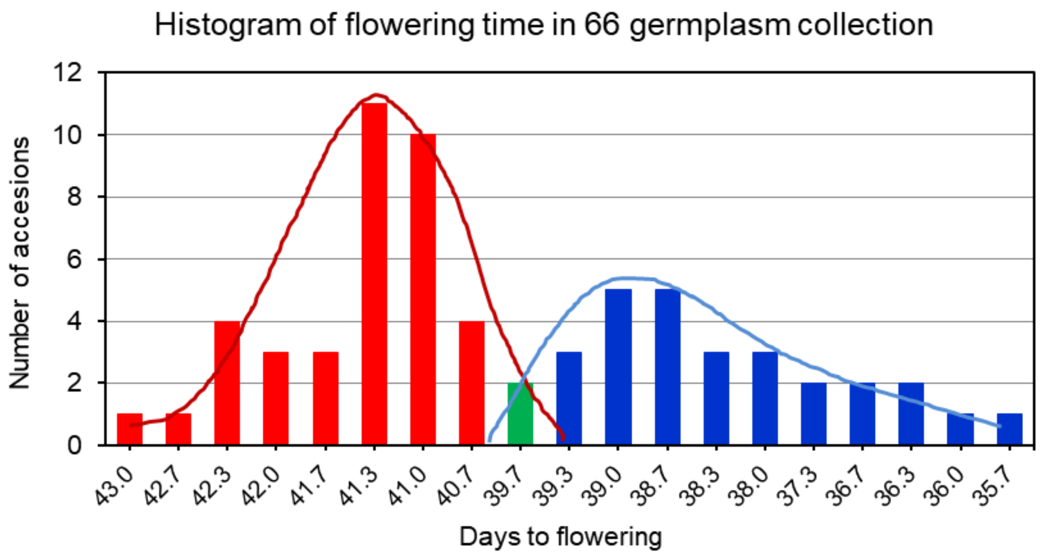

Red bars and line designate late-flowering accessions. Blue bars and line represent early-flowering accessions. Two accessions in the middle shown in green denote genotypes with ‘intermediate’ flowering time.

Supplementary material S8 is present in Excel-file.

Supplementary material S9. Results of Kruskal-Wallis test for the comparison between genotyping of three groups of genotypes (*aa*, *ab* and *bb*) with corresponding phenotyping results of lentil accessions for FT presented in Table 2.

	A	B	C	D	E	F	G	H
1	This spreadsheet performs the **Kruskal-Wallis test**. If you have just two groups, this is equivalent to a M							
2	It will handle up to 20 groups with up to 1000 observations in each group.							
3	For more information, see http://www.biostathandbook.com/kruskalwallis.html							
4	The spreadsheet comes with the oyster Fst data from that web page entered as an example.							
5	To use it, replace the oyster data with your numbers.							
6	"Adjusted H" is the test statistic, including an adjustment for the number of ties.							
7								
8								
11		**adjusted H:**	**54.163**					
12		**d.f.:**	**2**					
13		**P value:**	**1.73E-12**					
14								
15								
20	group names-->	aa	ab	bb				
21	data-->	43.7	41.0	39.3				
22		42.7	41.0	39.3				
23		42.3	40.7	39.3				
24		42.3	40.7	39.0				
25		42.3	40.7	39.0				
26		42.3	40.7	39.0				
27		42.0	39.7	39.0				
28		42.0	39.7	39.0				
29		42.0	39.0	38.7				
30		41.7		38.7				
31		41.7		38.7				
32		41.7		38.7				
33		41.3		38.7				
34		41.3		38.3				
35		41.3		38.3				
36		41.3		38.3				
37		41.3		38.0				
38		41.3		38.0				
39		41.3		38.0				
40		41.3		37.3				
41		41.3		37.3				
42		41.3		36.7				
43		41.3		36.7				
44		41.0		36.3				
45		41.0		36.3				
46		41.0		36.0				
47		41.0		35.7				
48		41.0						
49		41.0						
50		41.0						
51		41.0						
52								
